# Variations of Indole Metabolites and NRPS-PKS Loci in Two Different Virulent Strains of *Xenorhabdus hominickii*

**DOI:** 10.3389/fmicb.2020.583594

**Published:** 2020-11-24

**Authors:** Md. Mahi Imam Mollah, Miltan Chandra Roy, Doo-Yeol Choi, Md. Ariful Hasan, Md. Abdullah Al Baki, Hyun-Suk Yeom, Yonggyun Kim

**Affiliations:** ^1^Department of Plant Medicals, College of Life Sciences, Andong National University, Andong, South Korea; ^2^Center for Eco-Friendly New Materials, Korea Research Institute of Chemicals Technology, Daejeon, South Korea

**Keywords:** *Xenorhabdus hominickii*, *Lrp*, NRPS, PKS, secondary metabolite, eicosanoids

## Abstract

*Xenorhabdus hominickii* ANU1 is known to be an entomopathogenic bacterium symbiotic to nematode *Steinernema monticolum*. Another bacterial strain *X. hominickii* DY1 was isolated from a local population of *S. monticolum*. This bacterial strain *X. hominickii* DY1 was found to exhibit high insecticidal activities against lepidopteran and coleopteran species after hemocoelic injection. However, these two *X. hominickii* strains exhibited significant variations in insecticidal activities, with ANU1 strain being more potent than DY1 strain. To clarify their virulence difference, bacterial culture broths of these two strains were compared for secondary metabolite compositions. GC-MS analysis revealed that these two strains had different compositions, including pyrrolopyrazines, piperazines, cyclopeptides, and indoles. Some of these compounds exhibited inhibitory activities against phospholipase A_2_ to block eicosanoid biosynthesis and induce significant immunosuppression. They also exhibited significant insecticidal activities after oral feeding, with indole derivatives being the most potent. More kinds of indole derivatives were detected in the culture broth of ANU1 strain. To investigate variations in regulation of secondary metabolite production, expression level of leucine-responsive regulatory protein (*Lrp*), a global transcription factor, was compared. ANU1 strain exhibited significantly lower *Lrp* expression level than DY1 strain. To assess genetic variations associated with secondary metabolite synthesis, bacterial loci encoding non-ribosomal protein synthase and polyketide synthase (NRPS-PKS) were compared. Three NRPS and four PKS loci were predicted from the genome of *X. hominickii*. The two bacterial strains exhibited genetic variations (0.12∼0.67%) in amino acid sequences of these NRPS-PKS. Most NRPS-PKS genes exhibited high expression peaks at stationary phase of bacterial growth. However, their expression levels were significantly different between the two strains. These results suggest that differential virulence of the two bacterial strains is caused by the difference in *Lrp* expression level, leading to difference in the production of indole compounds and other NRPS-PKS-associated secondary metabolites.

## Introduction

Gram-negative and motile bacteria *Xenorhabdus* spp. belong to the family of Morganellaceae in the order of Enterobacterales. They share mutualistic relationship with entomopathogenic nematodes (EPNs) belong to genus *Steinernema* (Thomas and Poinar, 1979; Forst and Clarke, 2002). Mutualistic symbionts of EPNs have a similar life cycle: a phoretic form in the nematode host, a pathogenic form in the insect body, and a saprophytic form in the insect cadaver (Herbert and Goodrich-Blair, 2007; Stock and Goodrich-Blair, 2008; Koppenhofen and Gaugler, 2009). These symbiotic bacteria are localized in a specialized vesicle in the anterior part of the gut called receptacle (Snyder et al., 2007) of infective juveniles (IJs) at the third instar larval stage. Soil-dwelling IJs can infect and enter insect hemocoel (Akhurst, 1983). In the hemocoel, IJs will release these symbiotic bacteria that can suppress insects’ immune defense and induce septicemia to kill target insects (Dunphy, 1994). Nematodes can then grow and reproduce in the cadaver with nutrients supplied by their symbiotic bacteria through digestion of insect tissues with bacterial enzymes. When nutrients within the insect cadaver are consumed and nematode density reaches a carrying capacity, nematodes will develop into IJs and exit the cadaver to repeat their life cycle (Popiel et al., 1989).

Septicemia induced by *Xenorhabdus* or *Photorhabdus* is a main pathogenicity of EPNs (Park and Kim, 2000). However, there are virulence variations among different species of entomopathogenic bacteria (Sajjadian and Kim, 2020). The inter-specific variation in bacterial pathogenicity has been explained by variations in their immunosuppressive activities (Ahmed and Kim, 2018). Virulence variation in a species of *X. nematophila* has also been reported, showing that six strains have more than twofold difference in their insecticidal activities (Hasan et al., 2019). The intra-specific variation of virulence has also been explained by difference in immunosuppression (Hasan et al., 2019). Park et al. (2017) have explained that the difference in virulence is due to a phase variation of *X. nematophila* with respect to the expression of a specific virulence factor. There are two phenotypes in phase variation of *X. nematophila*: phase I (primary form) and phase II (secondary form). In phase I, but not in phase II, bacteria can produce antimicrobial substances such as indole derivatives, trans-stilbenes (Paul et al., 1981; Richardson et al., 1988), xenorhabdins, xenocoumacins (Mclnerny et al., 1991), and toxic proteinase (Kucera and Mracek, 1989; Balcerzak, 1991).

*Xenorhabdus* bacteria in insect hemocoel secrete several virulence factors to suppress insect immunity and kill target insects (Sergeant et al., 2006; Shrestha and Kim, 2007). For example, *X. nematophila* can secrete at least eight secondary metabolites to suppress insect immunity by inhibiting phospholipase A_2_ (PLA_2_) activity (Seo et al., 2012). In addition, *Xenorhabdus* bacteria can secrete toxin proteins including Xpt (Morgan et al., 2001), Txp40 (Brown et al., 2006), XaxAB (Vigneux et al., 2007), XnGroEL (Kumari et al., 2014), PirAB (Yang et al., 2017), and a 12 kDa protein (Hemalatha et al., 2018). These bacterial products can induce fatal septicemia and toxemia of target insects (Tobias et al., 2017; Shi and Bode, 2018).

Secondary metabolites produced by *Xenorhabdus* bacteria are associated with biosynthetic gene clusters (BGCs) including non-ribosomal peptide synthetase (NRPS) and polyketide synthase (PKS) (Tobias et al., 2017). Predicted and identified compounds from NRPS-PKS are associated with bacterial pathogenicity (Shi and Bode, 2018). For example, an isocyanide-containing compound rhabducin produced from BGC consisting of *isnA*, *isnB*, and a glycosyltransferase can inhibit the activity of phenoloxidase (PO) in the wax moth, *Galleria mellonella* (Crawford et al., 2012). More than 70 kinds of rhabdopeptide/xenortide peptides (RXPs) have been derived from BGCs encoding multiple NRPS subunits. They are structurally similar to protease inhibitors. They can suppress PO activation (Cai et al., 2016; Sussmuth and Mainz, 2017). Phurealipids produced from NRPS/PKS can prevent expression of antimicrobial peptide (AMP) genes (Nollmann et al., 2015). With respect to controlling gene expression related to bacterial pathogenicity, a global transcription factor such as leucine-rich protein (*Lrp*) plays a crucial role in virulence modulation. Low *Lrp* levels are associated with immunosuppression in insect hosts (Casanova-Torres et al., 2017) by producing secondary metabolites that can inhibit AMP gene expression. Thus, genetic factors potentially associated with virulence variations of *Xenorhabdus* bacteria have been well documented. However, whether genetic variation of NRPS-PKS and Lrp known to mediate secondary metabolites is associated with virulence variation of entomopathogenic bacteria remains unclear.

This study compared secondary metabolites produced by two *X. hominickii* strains with different insecticidal activities. *Lrp* expression levels in these two bacteria strains were also compared to understand overall control of gene expression associated with bacterial virulence. Finally, genetic variations in NRPS-PKS gene clusters between the two strains were analyzed to explain the variation in gene products.

## Materials and Methods

### Insect Rearing

Larvae of beet armyworm, *Spodoptera exigua*, were collected from Welsh onion (*Allium fistulsum* L.) fields in Andong, Korea and reared with an artificial diet (Goh et al., 1990). Larvae of *Maruca vitrata* were collected from adzuki bean (*Vigna angularis*) in Suwon, Korea and reared with an artificial diet (Jung et al., 2009). Mealworm (*Tenebrio molitor*) larvae were provided by Bio Utility, Inc. (Andong, Korea) and reared with wheat bran supplemented with cabbage (Liu et al., 2020). Rearing conditions were maintained at a temperature of 25 ± 2°C, relative humidity of 60 ± 5%, and a photoperiod of 16:8 h (L:D). Adults were provided with 10% sucrose solution.

### Chemicals

Indole, tryptophan, tryptophol, indole-3-acetic acid hydrazide, and 5-methyl-2-phenyl indole were purchased from Sigma-Aldrich Korea (Seoul, Korea) and dissolved in dimethylsulfoxide (DMSO). Two different PLA_2_ substrates, 1,2-bis (heptanoylthio) phosphatidylcholine (CP) for secretory PLA_2_ (sPLA_2_) activity and arachidonoyl thio-PC for cellular PLA_2_ (cPLA_2_) activity, were purchased from Cayman Chemical (Ann Arbor, MI, United States). Peptides cyclo-(l-leucyl-l-phenylalanyl) (cLF) and cyclo-(glycyl-l-leucyl) (cGL) were chemically synthesized. *N*-acetyl tryptamine (NAT) was purchased from Cayman Chemical. 3-Methyl-6-(phenylmethyl)-2,5-piperazinedione (MPP) was purchased from ChemFaces (Wuhan, Hubei, China). 3-Phenylmethyl-2,5-piperazinedione (PMP) was purchased from Toronto Research Chemicals (North York, Canada). *N*-(2-phenylethyl) acetamide (NPA) was kindly provided by Professor Helge Bode (Goethe University, Frankfurt, Germany). Hexahydro-pyrrolo[1,2-a]pyrazine-1,4-dione (HPA), hexahydro-3-(2-methylpropyl)-pyrrolo[1,2-a]pyrazine-1,4-dio ne (HMPP), and hexahydro-3-(phenylmethyl)- pyrrolo[1,2-a]pyrazine-1,4-dione (HPPP) were purchased from Interpharm (Koyang, Korea). Phosphate-buffered saline (PBS, pH 7.4) was prepared with 100 mM phosphoric acid. Anticoagulant buffer (ACB, pH 4.5) was prepared to contain 186 mM NaCl, 17 mM Na_2_EDTA, and 41 mM citric acid.

### Nematode Collection and Multiplication

A nematode colony collected from Jinju, Korea was obtained from Professor Dongwoon Lee (Kyungpook National University, Sangju, Korea). It was multiplied in the fifth instar larvae (L5) of *S. exigua*. In brief, 500 μL of distilled water containing about 1,000 IJs was topically applied to 5 larvae in a Petri dish (9 cm in diameter, 3 cm in height). Infected larvae were then incubated at 25 ± 2°C with an artificial diet for 3–5 days. Subsequent dead larvae were transferred to White traps (Lee et al., 2000). Emerging IJs were harvested daily and stored at 10°C for no more than 21 days before use (Park et al., 1998).

### Isolation of Symbiotic Bacteria

To isolate symbiotic bacteria from nematodes, approximately 200 IJs of nematodes were topically applied to L5 larvae of *S. exigua* and incubated at 25 ± 2°C for 12 h. Hemolymph from infested larvae was collected and streaked onto NBTA (nutrient agar supplemented with 25 mg bromothymol blue and 40 mg triphenyl tetrazolium chloride in 1 L) medium. Blue colored colonies of bacteria from NBTA plates were sub-cultured in tryptic soy broth (TSB) (Difco, Sparks, MD, United States) at 28°C for 48 h.

### Analysis of Nematode Morphological Characters

Morphological characters of IJs were used to identify nematodes. IJs were fixed in triethanolamine formalin using the method described by Seinhorst (1959). Glycerin solution was added to nematode slide to prevent desiccation. The slide was then observed under a phase contrast microscope (BX41, Olympus, Tokyo, Japan) to measure total body length, excretory pore size, and tail length. Morphological characters of IJs were then compared with those of *S. monticolum* (Stock et al., 1997). Each character measurement used 10 different IJs.

### Nematode DNA Extraction and Internal Transcribed Spacer (ITS) Sequence Analysis

Genomic DNA (gDNA) was extracted from 0.5 g of IJs according to Kang et al. (2005). The resulting gDNA was used as a template for PCR amplification of ITS using M13 universal primer sequence-linked ITS primers (5′-TTGATTACGTCCCTGCCCTTT-3′ and 5′-TTTCAC TCGCCGTTACTAAGG-3′) reported by Vrain et al. (1992). PCR was performed with 35 cycles of denaturation at 94°C for 1 min, annealing at 46°C for 1 min, and extension at 72°C for 1 min. DNAs were sequenced bidirectionally by Macrogen (Seoul, Korea) using M13 forward and reverse primers. BlastN program of the National Center for Biotechnology Information^1^ was used to analyze nucleotide sequence. Sequences were aligned using BioEdit 7.2.5 (Hall, 1999). Evolutionary relationship was inferred with MEGA6 program using the Neighbor-Joining method (Tamura et al., 2013). Bootstrapping values on branches were obtained with 1,000 repetitions.

### Carbon Utility Analysis of the Bacteria Using a Biolog System

Biochemical tests of isolated bacterial clones followed the method described previously (Park et al., 2017). Resulting characters were used to determine bacterial genus by comparing them with those of bacteria described in Bergey’s Manual (Krieg and Hart, 1984). Acid producing characters of isolates using different carbon sources were assessed with a colorimetric method using a GEN III microplate (Biolog, Hayward, CA, United States) and compared with characters of different *Xenorhabdus* species.

### Examination of Bacterial Isolate Using Transmission Electron Microscope (TEM)

Overnight grown bacteria were centrifuged at 10,000 × *g* for 20 min. Cell pellet was resuspended and washed three times with PBS. Bacteria suspension was diluted with deionized and distilled (ddH_2_O). After attaching bacteria onto 200 mesh copper grid coated with carbon-stabilizer formvar and repeatedly rinsing with ddH_2_O followed by drying, bacteria were negatively stained with 2% phosphotungstic acid. Each sample was then observed with a TEM EM 900 T (Zeiss, Oberkochen, Germany) with 12,000× to 80,000× magnifications.

### Bacterial DNA Extraction and 16S rRNA Sequence Analysis

To identify the symbiont bacterium, genomic DNA was extracted using a QIA prep Spin Miniprep kit (Qiagen, Valencia, CA, United States). The 16S rRNA region was amplified by PCR using forward and reverse primers of 5′-AGAGTTTGATCCTGGCTCAG-3′ and 5′-GGCTACCTTGTTACGACTT-3′ reported by Eden et al. (1991). PCR amplification was performed using 35 cycles of denaturation at 94°C for 1 min, annealing at 50°C for 1 min, and extension at 72°C for 1 min. The resulting PCR product was bidirectionally sequenced. The nucleotide sequence obtained was assessed with the same method describe earlier for nematode identification.

### Bacterial Virulence Test

For pathogenicity tests, 24 h-cultured bacterial cells were washed three times with sterilized PBS by centrifuging at 4,000 × *g* for 2 min at 4°C. Cell pellet was re-suspended in PBS. For dose-mortality assay, freshly cultured bacteria at different concentrations (0, 10^1^, 10^2^, 10^3^, 10^4^, 10^5^ and 10^6^ CFU/larva) were injected into insect hemocoels using 10-μL Hamilton microsyringes (Hamilton, Reno, NV, United States) after surface-sterilization with 70% ethanol. Test larvae used L5 stages of *S. exigua* and *M. vitrata*. For *T. molitor*, 3-cm body size larvae were used. Mortality was observed at 24 h after bacterial injection. *Escherichia coli* Top10 (Invitrogen, Seoul, Korea) was used as control bacteria for treatment. Each treatment consisted of three replications. Each replication of bacterial concentration used 10 larvae.

### Extraction of Bacterial Secondary Metabolites and Thin Layer Chromatography (TLC)

Each bacterial strain was cultured separately in 1 L of TSB at 28°C for 48 h. The cultured broth was centrifuged at 10,000 × *g* for 20 min at 4°C to obtain supernatant which was then subjected to fractionation. Briefly, the same volume (1 L) of hexane was mixed with the supernatant to separate organic and aqueous fractions. The resulting aqueous fraction was combined with the same volume of ethyl acetate. These processes were sequentially repeated for chloroform and butanol organic solvents. Resulting organic extracts [hexane extract (HEX), ethyl acetate extract (EAX), chloroform extract (CX), and butanol extract (BX)] containing bacterial metabolites were dried with a rotary evaporator (Eyela N-1110, Rikakikai, Tokyo, Japan) at 20°C for HEX, 25°C for CX, 30°C for EAX, and 40°C for BX. After weighing dried metabolites, each extract was resuspended with 5 mL of methanol. Resulting metabolites were subjected to TLC using silica gel plates (20 cm × 20 cm; Merck, Darmstadt, Germany). After developing with chloroform:methanol:acetic acid (7:2.5:0.5, v/v) as an eluent, silica gel plates were incubated with a mixture (19:1, g/g) of sea sand (Merck) and iodine (Duksan, Ansan, Korea). Spots were then visualized in a fluorescence analysis cabinet (Spectroline, CM-10, Westbury, NY, United States).

### Secondary Metabolite Analysis Using Gas Chromatography-Mass Spectrometer (GC-MS)

To identify compounds in organic extracts, GC-MS analysis was carried out using a GC (7890B, Agilent Technologies, Santa Clara, CA, United States) equipped with MS (5977ANetwork, Agilent Technologies). The GC was also equipped with an HP5 MS column (non-polar column, Agilent Technologies) with an internal diameter of 30 m × 250 μM and a film thickness of 0.25 μm. Helium was used as a carrier gas at a flow rate of 1 mL/min. Injector temperature was set at 200°C at a split mode with a split ratio of 10:1. Initially, oven temperature was set to be 100°C for 3 min and then raised to 300°C at a rate of 5°C/min. This oven temperature was then retained for 10 min. Total running time was 53 min. Mass spectra were recorded in EI mode at 70 eV with a scanning range of 33–550 m/z. Purified samples were respectively identified based on mass spectra of compounds compared to those deposited in the database of NIST11 (U.S. Department of Commerce, Gaithersburg, MD, United States) and literature data^2^.

### Prediction of NRPS-PKS Loci From the Genome of *X. hominickii* ANU1

From the whole genome sequence of *Xenorhabdus hominickii* ANU1 (GenBank accession number: CP016176.1), NRPS-PKS loci were predicted. Specific gene clusters in these NRPS-PKS loci were subjected to NRPS/PKS analysis^3^ and confirmed with pfam^4^.

### Sequencing of NRPS-PKS Loci of *X. hominickii* DY1 Strain

NRPS-PKS sequences of *X. hominickii* DY1 were read in a chromosomal walking manner using sequential primers for each specific region (Supplementary Table 1). PCR amplification was performed using 35 cycles of denaturation at 94°C for 1 min, annealing at appropriate temperature based on primers, and extension at 72°C for various time based on primers. Resulting PCR products were bidirectionally sequenced.

### Hemocyte Nodule Formation Analysis

Hemocyte nodule formation in response to bacterial challenge was performed using 3-day old L5 larvae of *S. exigua* by injecting heat-killed bacteria (4 × 10^4^ cells/larva) into the hemocoel through prolegs. After incubation at 25°C for 8 h, larvae were dissected to count nodules under a stereomicroscope at 50× magnification. Each treatment consisted of three replicates. Each replicate used five larvae.

### PLA_2_ Enzyme Activity Measurement

Activities of two different types of PLA_2_, secretory PLA_2_ (sPLA_2_) and cytosolic PLA_2_ (cPLA_2_), were measured according to the method described by Vatanparast et al. (2018).

### Reverse Transcription and Quantitative Polymerase Chain Reaction (RT-qPCR)

Total RNAs were extracted from bacteria using Trizol reagent (Ambion, Carlsbad, CA, United States) according to the manufacturer’s instructions. First-strand cDNAs were then synthesized from total RNAs using iScript Select cDNA Synthesis Kit with random primer (Bio-Rad, Alfred Nobel Drive Hercules, CA, United States) according to the manufacturer’s instructions. Synthesized cDNAs were used as templates for RT-PCR and RT-qPCR. qPCR was performed at 95°C for 4 min for initial denaturation followed by 40 cycles of 95°C for 30 s, 52°C for 30 s, and 72°C for 30 s. Two ribosomal genes, *RL32* and *16S rRNA*, were used as reference genes for bacteria samples grown in insects or with culture media. Quantitative analysis was done with a comparative CT method (Livak and Schimittgen, 2001) to estimate mRNA expression levels. Each experiment was replicated three times.

### Statistical Analysis

All data for continuous variables were subjected to one-way analysis of variance (ANOVA) using PROG GLM in SAS program (SAS Institute Inc, 1989). Mortality data were subjected to arcsine transformation and used for ANOVA. Means were compared with the least significant difference (LSD) test at Type I error = 0.05. Median lethal dose (LD_50_) was subjected to Probit analysis using EPA Probit Analysis Program ver. 1.5 (U.S. Environmental Protection Agency, Washington, DC, United States).

## Results

### Identification of an EPN Isolate

**FIGURE 1:**
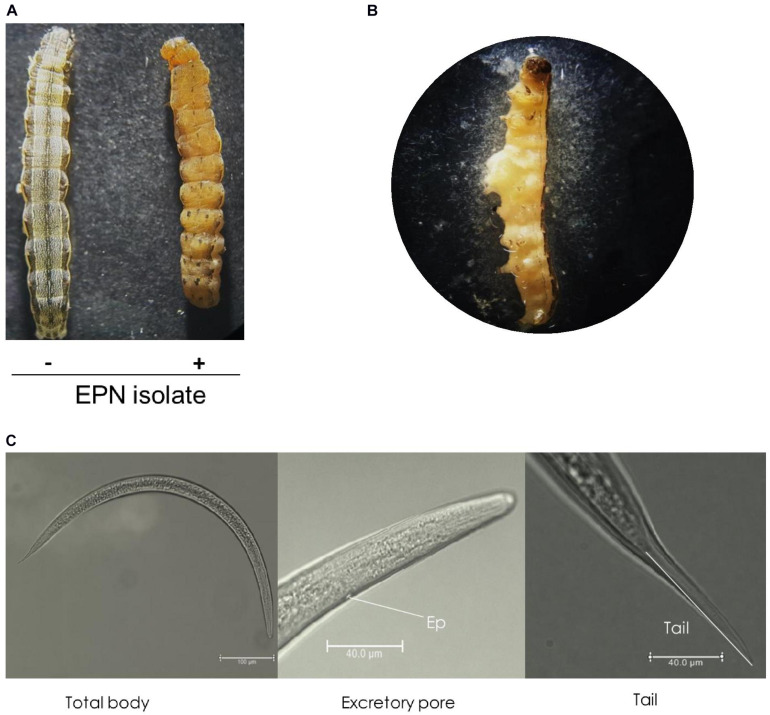
An entomopathogenic nematode (EPN) isolate multiplied in *S. exigua* host. **(A)** Symptom of a dead insect larva infected (+) with the nematode isolate compared to an uninfected (−) healthy larva. **(B)** Release of infective juveniles (IJs) from the dead larva. **(C)** Morphological characters of IJs, including total body length from a whole view, lateral view showing excretory pore (‘Ep’), and terminal view showing tail. IJs were topically applied onto L5 larvae of *S. exigua*. IJs were fixed in triethanolamine formalin for observation under a phase contrast microscope.

An ENP isolate was infective to larvae of *S. exigua*. It killed them after topical application. Dead insects did not emit any bioluminescence. However, they showed a pale brown color in 7–10 days (Figure 1A), suggesting a species of *Steinernema*. When the hemocoel was open, reproduced IJs were released (Figure 1B). These IJs were used to identify the nematode using morphological characters such as total body length, excretory pore size, and tail length (Figure 1C). When these morphological characters of the IJs were compared with those of *Steinernema* spp., they matched to characters of *S. monticolum* (Supplementary Table 2).

**FIGURE 2:**
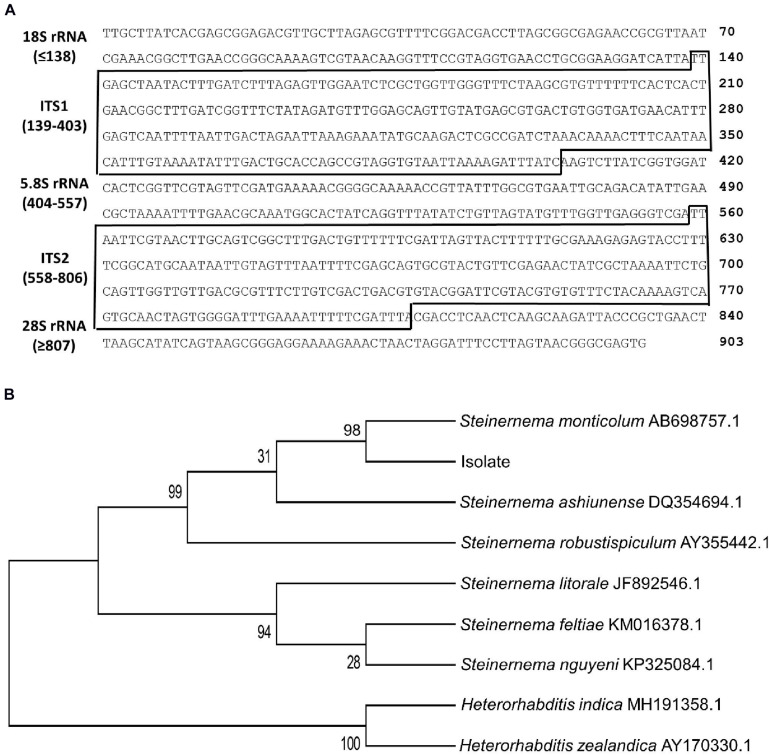
Molecular identification of an EPN isolate using ITS sequence (GenBank accession number: AF122017.1). **(A)** Two ITS sequences (‘ITS-1’ and ‘ITS-2’) between neighboring ribosomal RNA (rRNA) sequences. These two ITS regions are marked with boxes. **(B)** Phylogeny analysis of EPN isolate with other EPNs. The tree was constructed with the Neighbor-joining method using MEGA6. Bootstrapping values on branches were obtained with 1,000 repetitions. GenBank accession numbers of ITS/rRNA sequences follow their own species names.

To support such morphological identification, ITS of rRNA genes from the isolate was sequenced. The sequence (903 nucleotides, Figure 2A) comprised of partial 18S rRNA, ITS-I, 5.8S rRNA, ITS-2, and partial 28S rRNA after aligning with the corresponding sequence of *Caenorhabditis elegans* (Ellis et al., 1986). Based on known ITS sequences of *Steinernema* spp., this isolate shared the highest sequence identities (98%) with *S. monticolum*. Phylogeny tree analysis showed that the isolate was separated from *Heterorhabditis* and other *Steinernema* spp. However, it was clustered with known *S. monticolum* (Figure 2B).

### Identification of a Symbiotic Bacterium of *S. monticolum*

**FIGURE 3:**
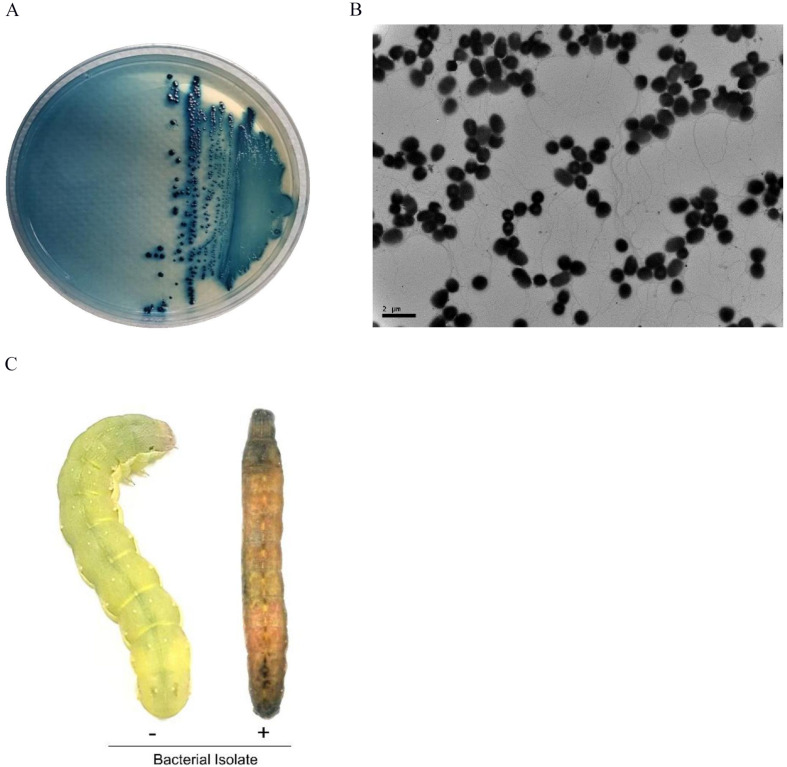
A bacterial isolate from *S. monticolum*. **(A)** Blue colonies on NBTA plate medium. **(B)** TEM photo of the bacterium showing flagella. Scale bar, 2 μm. **(C)** Symptom of a dead larva infected with the bacterial isolate. From NBTA plate, single blue colonies were sub-cultured into TSB media. Overnight grown bacteria were injected into L5 larvae of *S. exigua* for pathogenicity analysis.

Due to a well-known relationship between EPN and symbiotic bacteria, hemolymph of *S. exigua* larvae infected with *S. monticolum* was collected and plated onto NBTA medium to culture bacteria. Blue colonies were obtained on the medium (Figure 3A) and used to examine bacterial ultrastructure using TEM (Figure 3B). Rod shape bacteria (length: 1.30 ± 0.20 μm; width: 1.02 ± 0.11 μm) with flagella were seen. The blue colony was entomopathogenic to *S. exigua* larvae (Figure 3C).

Isolated blue colonies from NBTA plates did not show catalase activity or bioluminescence emission (Supplementary Table 3), indicating that it belonged to *Xenorhabdus*, but not to *Photorhabdus.* These two genera are known to be associated with EPNs. In addition, carbon utilization characters (Supplementary Table 4) of these bacteria were more similar (96.7%) to those of *X. hominickii* than to other *Xenorhabdus* spp. This identification results using biochemical characters was further supported by 16S rRNA sequence analysis (Supplementary Figure 1A). Sequence alignment of its 16S rRNA with 16S rRNA sequences of different *Xenorhabdus* spp. indicated that this isolate had the highest similarity (100%) with *X. hominickii* ANU1 strain (NCBI GenBank accession number: CP016176.1). In phylogenetic analysis, this bacterial isolate from *S. monticolum* was clustered with *X. hominickii* (Supplementary Figure 1B). Based on these biochemical and molecular identifications, this bacterial isolate was named as *X. hominickii* DY1 strain.

### Virulence Comparison of Two *X. hominickii* Strains

**FIGURE 4:**
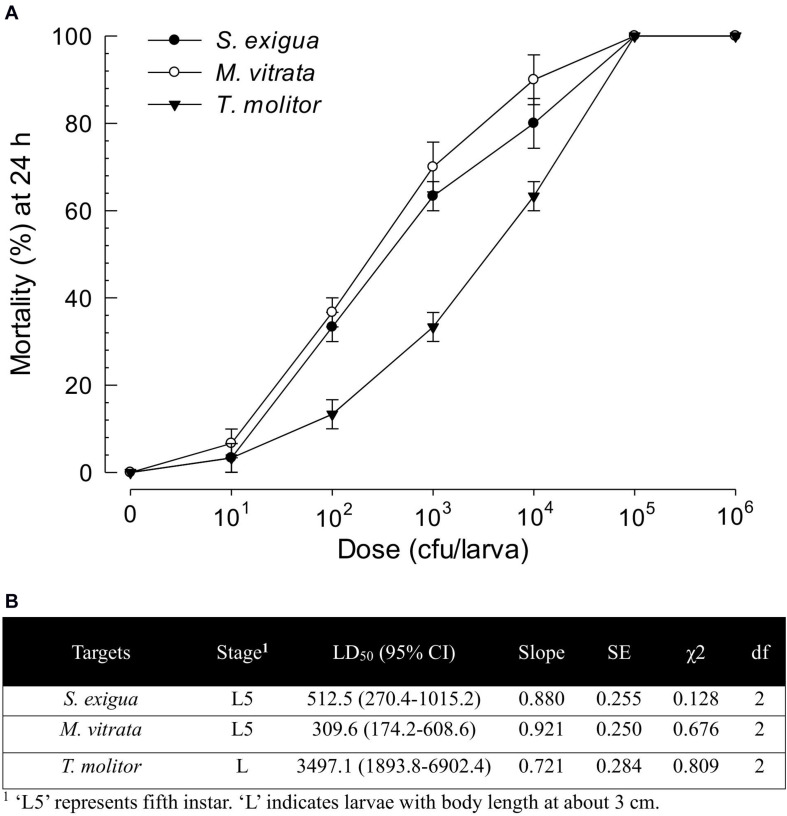
Virulence of *X. hominickii* DY1 against different insects of two lepidopteran species (*S. exigua* and *P. xylostella* at L5 larval stage) and one coleopteran species (*T. molitor* at larval stage with 3-cm body length). **(A)** Dose-mortality curve at 24 h after bacterial infection. Overnight cultured bacteria were injected into hemocoels of surface-sterilized larvae using microsyringes. **(B)** Medium lethal dose (LD_50_). LD_50_ values are expressed as colony forming unit (cfu) of bacteria per larva. Each treatment was replicated three times. Each replication used 10 larvae.

*X. hominickii* DY1 strain exhibited insecticidal activities against lepidopteran and coleopteran species in a dose-dependent manner (Figure 4A). Only 100 live bacteria were enough to show significant (*P* < 0.05) pathogenicity against two lepidopteran species (*S. exigua* and *M. vitrata*). However, its pathogenicity was much lower against *T. molitor*, a coleopteran species. Its LD_50_ value (bacterial dose) against *T. molitor* was more than 10-fold than that for *M. vitrata* (Figure 4B).

**FIGURE 5:**
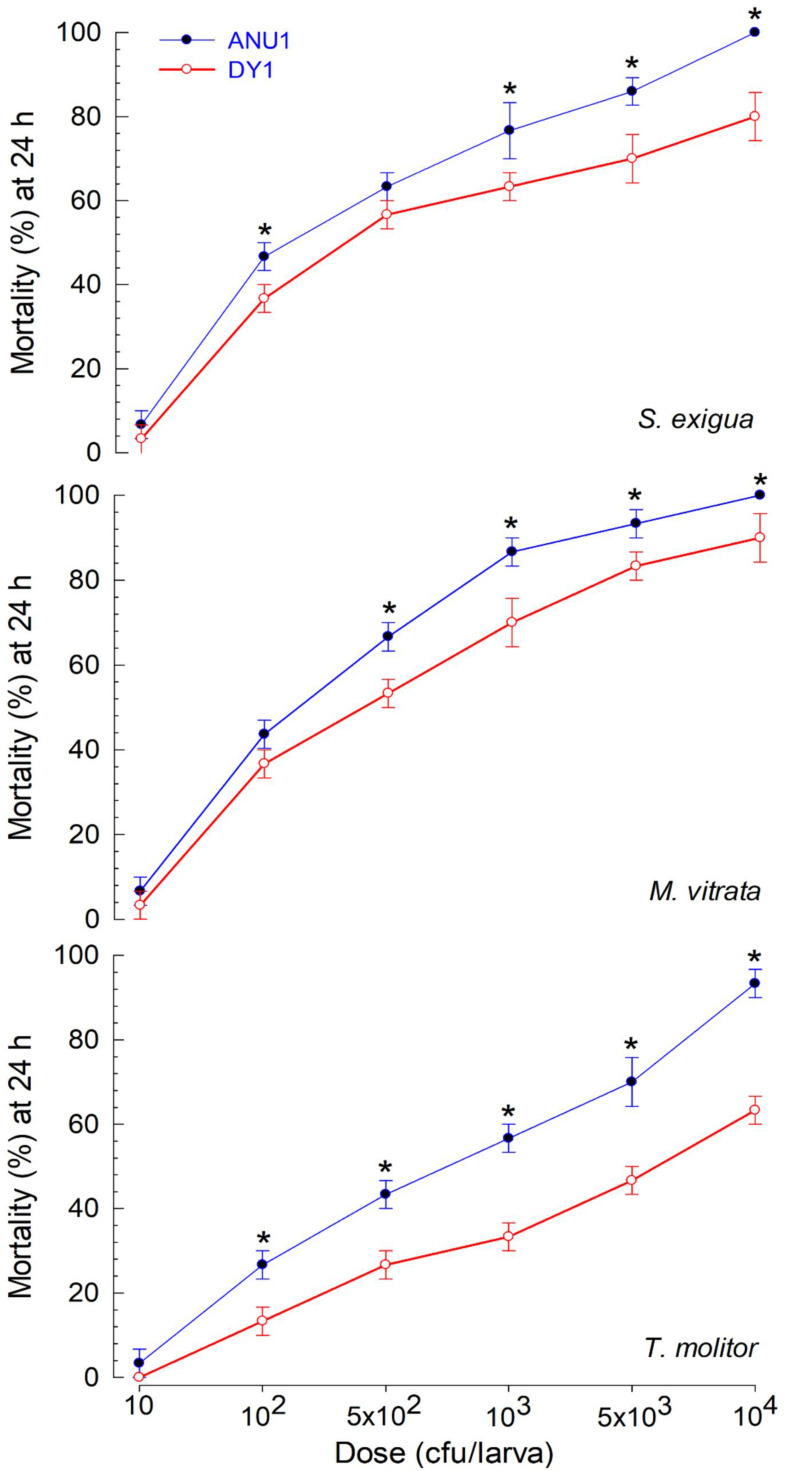
Differential virulence of two *X. hominickii* strains (‘ANU1’ and ‘DY1’) against two lepidopteran species (*S. exigua* and *M. vitrata* at L5 larval stage) and a coleopteran species (*T. molitor* at larval stage with 3-cm body length). Overnight cultured bacteria were injected into hemocoels of surface-sterilized larvae using microsyringes. Each treatment was replicated three times. Each replication used 10 larvae. Asterisks above standard error bars indicates significant differences between means of two strains for each dose at Type I error = 0.05 (LSD test).

Two *X. hominickii* strains of ANU1 (Park et al., 2017) and DY1 were compared in virulence against common target insects (Figure 5). For all three insect targets, ANU1 was more virulent than DY1 based on LD_50_ values (228.1 versus 502.1 cfu/larva against *S. exigua*, 152.3 versus 322.6 cfu/larva against *M. vitrata*, and 666.8 versus 3,573.3 cfu/larva against *T. molitor*).

### Variation in Secondary Metabolites Between Two Bacterial Strains

To clarify the differential virulence of the two strains of *X. hominickii*, secondary metabolites released into culture media were compared using organic extracts. Four organic solvents (hexane, ethyl acetate, chloroform, and butanol) were sequentially used to extract bacterial metabolites. These metabolite extracts were subjected to TLC analysis (Supplementary Figure 2A). Total TLC spot numbers of ANU1 extracts were more than those of DY1 extracts except butanol extract (Supplementary Figure 2B). To predict secondary metabolites, these extracts were analyzed using GC-MS (Supplementary Table 5). A total of 121 compounds were predicted from all four organic extracts. Almost three quarters (72.7%) of compounds were extracted with hexane and ethyl acetate while the remaining 33 compounds were extracted only with chloroform and butanol. Total numbers of compounds identified in the bacterial culture broth were different between two strains (93 compounds from ANU1 vs. 57 compounds from DY1).

### Indole Derivatives and Their Biological Activities

**FIGURE 6:**
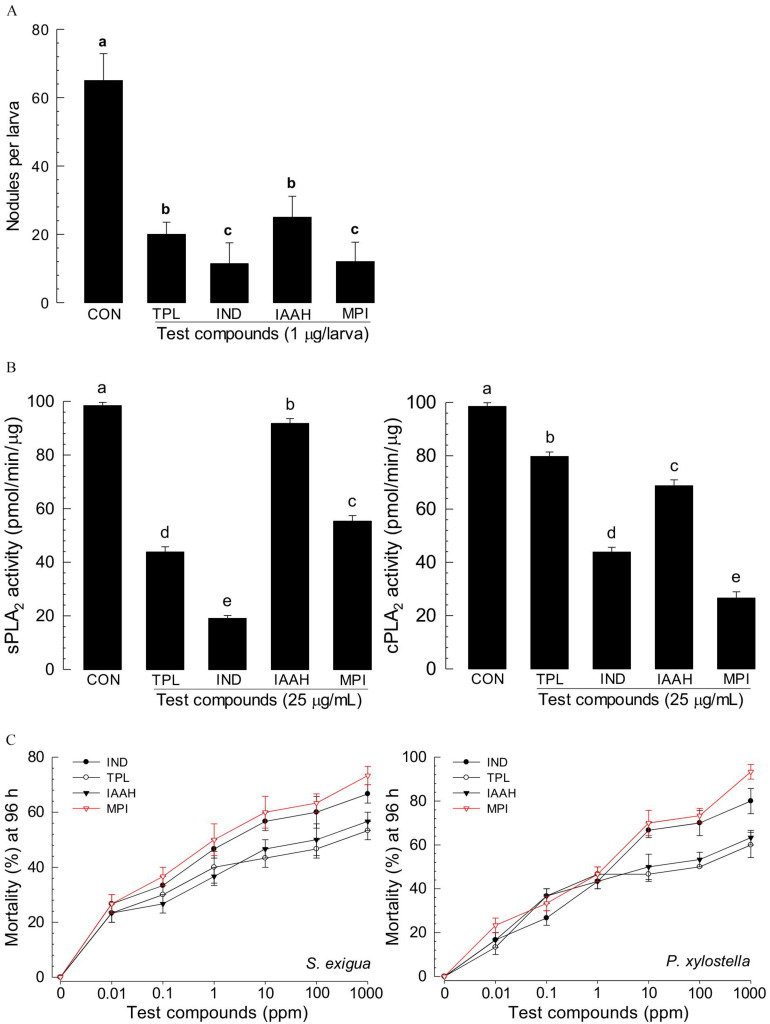
Immunosuppressive and insecticidal activities of indole and its derivatives identified from *X. hominickii* culture broth. Acronyms of test compounds are described in Table 1. **(A)** Assessment of immunosuppressive activities using nodulation assay. Test compounds (1 μg/larva) were injected to L5 larvae of *S. exigua* along with *E. coli* (10^4^ cfu/larva) using microsyringes. **(B)** Their inhibitory activities against hemocyte sPLA_2_ and cPLA_2_ of L5 larvae. Each treatment was replicated three times with independent samplings. Control (‘CON’) used DMSO solvent that was used for diluting test compounds. Each treatment was assessed using five larvae. Different letters above standard error bars indicate significant differences among means at Type I error = 0.05 (LSD test). **(C)** Their insecticidal activities against L3 larvae of *S. exigua* and *P. xylostella* using a leaf dipping method. Each treatment was replicated three times. Each replication used 10 larvae.

Among differentially produced secondary metabolites, indole derivatives were clearly distinct between two bacterial strains (Table 1). Five indole derivatives were detected from ANU1 strain while only indole was produced by DY1. Indole and three derivatives significantly (*P* > 0.05) suppressed a cellular immune response based on nodule formation against bacterial infection (Figure 6A). Among them, indole and methyl phenyl indole (MPI) had the most potent activities. These immunosuppressive activities of four indole compounds were explained by their inhibitory activities against sPLA_2_ and cPLA_2_. Among four indole compounds, indole and MPI showed the highest inhibitory activities against sPLA_2_ and cPLA_2_, respectively (Figure 6B). Insecticidal bioassay using leaf-dipping method showed that these four indole derivatives had potent insecticidal activities, with indole and MPI being the most potent (Figure 6C).

**TABLE 1:** Prediction of indole compounds produced by *X. hominickii* ANU101 (‘ANU’) and *X. hominickii* DY1 (‘DY’).

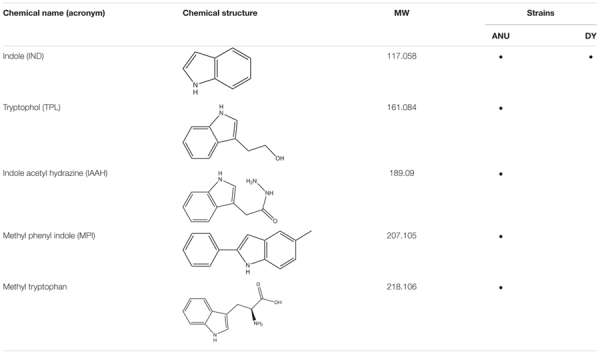

### NRPS-PKS Derivatives and Their Biological Activities

Among secondary metabolites produced by *X. hominickii*, production of 12 compounds might be influenced by NRPS-PKS catalytic activities because they contained peptides and polyketides (Table 2). These 12 compounds included three pyrrolopyrazines, four piperazines, three peptides, and two amines. Although pyrrolopyrazines were commonly produced by two *X. hominickii*, other metabolites were not.

**TABLE 2:** Prediction of secondary metabolites synthesized by NRPS-PKS of *X. hominickii* ANU101 (‘ANU’) and *X. hominickii* DY1 (‘DY’).

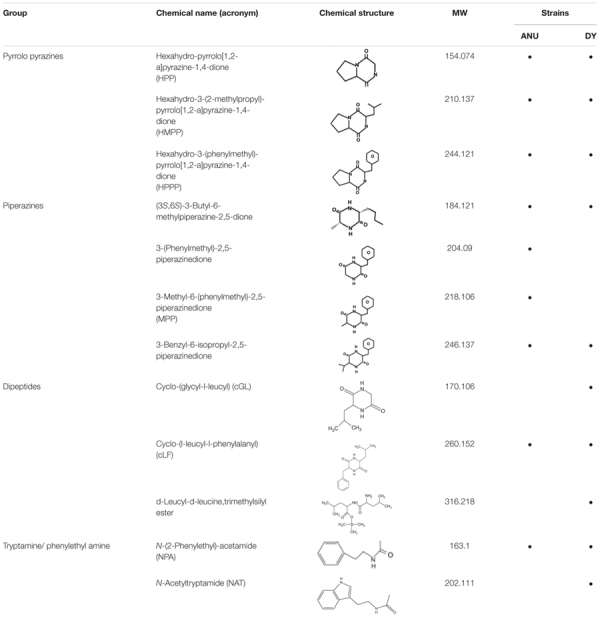

**FIGURE 7:**
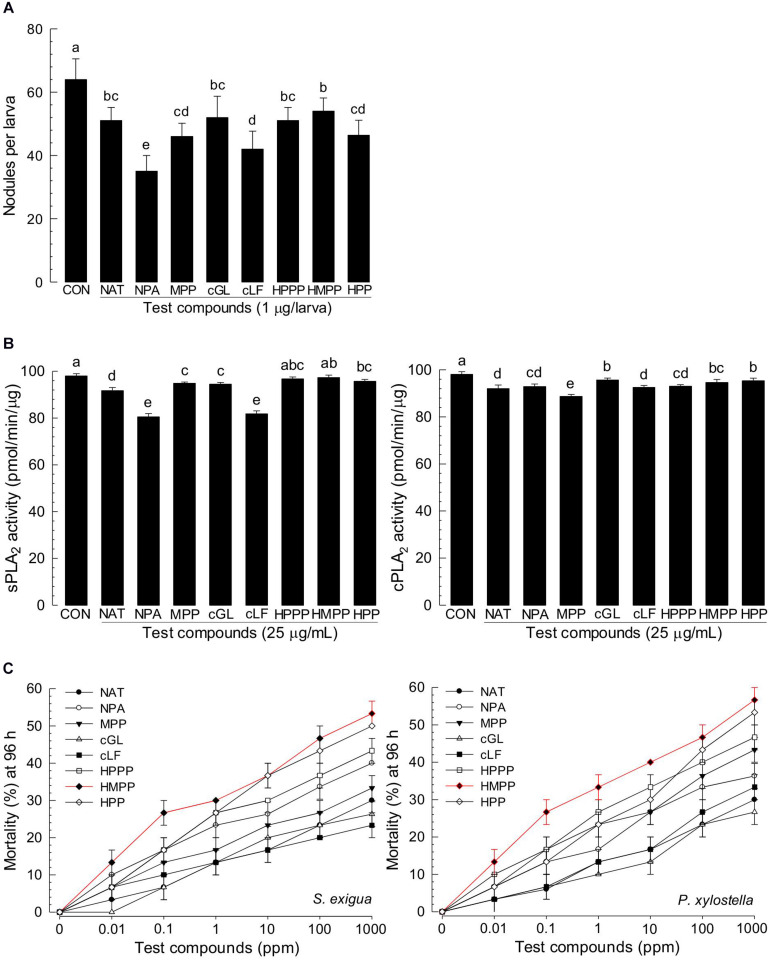
Immunosuppressive and insecticidal activities of NRPS-PKS-derived secondary metabolites identified from *X. hominickii* culture broth. Acronyms of test compounds are described in Table 2. **(A)** Assessment of immunosuppressive activities using nodulation assay. Test compounds (1 μg/larva) were injected to L5 larvae of *S. exigua* along with *E. coli* (10^4^ cfu/larva) using microsyringes. **(B)** Their inhibitory activities against hemocyte sPLA_2_ and cPLA_2_ of L5 larvae. Each treatment was replicated three times with independent samplings. Control (‘CON’) used DMSO solvent that was used for diluting test compounds. Each treatment was assessed using five larvae. Different letters above standard error bars indicate significant differences among means at Type I error = 0.05 (LSD test). **(C)** Their insecticidal activities against L3 larvae of *S. exigua* and *P. xylostella* using a leaf dipping method. Each treatment was replicated three times. Each replication used 10 larvae.

Eight products of NRPS-PKS were assessed for their immunosuppressive activities (Figure 7). All five metabolites significantly (*P* < 0.05) suppressed nodule formation (Figure 7A). These immunosuppressive activities were supported by their inhibitory activities against PLA_2_ (Figure 7B). All these compounds exhibited insecticidal activities after feeding administration. However, they were much less potent than indole derivatives (Figure 7C).

### Prediction of NRPS-PKS Loci and Variation in Amino Acid Sequences Between Two Bacterial Strains

**FIGURE 8:**
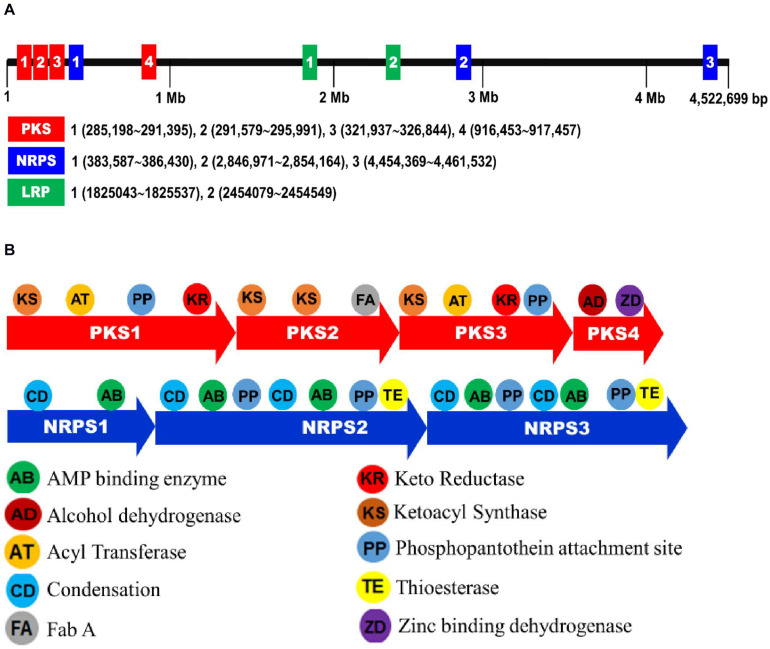
Biosynthetic gene clusters associated with secondary metabolites of *X. hominickii* ANU1. **(A)** A genome map indicating NRPS-PKS and Lrp loci of *X. hominickii* ANU1. **(B)** Component analysis of each NRPS-PKS locus.

From the genome of *X. hominickii* ANU1, three NRPS (NRPS1-NRPS3) and four PKS (PKS1-PKS4) loci were predicted (Figure 8). These seven loci were sequenced from DY1 strain and compared with those of ANU1 (Supplementary Figure 3). These two strains shared over 99% sequence identities for all seven loci. However, several point mutations were detected between these two strains (Table 3). With respect to amino acid sequences, these two strains showed genetic variations ranging from 0.12% for PKS3 to 0.67% for NRPS2. To assess expression levels of leucine-responsive regulatory protein (Lrp), a global transcription factor, two Lrp genes (*Lrp1* and *Lrp2*) were predicted from *X. hominickii* ANU1 strain (Supplementary Figure 4). They were localized between *PKS4* and *NRPS2*. These two Lrp genes showed 34.7% sequence homologies in their amino acid sequences. However, these two Lrp genes were separately clustered with other homologous Lrp genes of *Xenorhabdus* and *Photorhabdus* (Supplementary Figure 5).

**TABLE 3:** Variation in amino acid sequences of PKS and NRPS loci between ANU101 and DY1 strains of *X. hominickii*.

Loci	AA	Variations		Rate (%)
		#	Locations	
PKS1	2,065	11	168(M→I), 169(M→L), 219(D→N), 330(F→W), 410(A→G), 670(P→S), 871(T→A), 989(F→L), 1069(R→G), 1249(K→N), 1709(F→S)	0.53
PKS2	1,471	4	292(I→F), 491(D→G), 1012(Q→R), 1053(F→L)	0.27
PKS3	1,635	2	652(S→R), 1370(N→K)	0.12
PKS4	334	1	69(I→M)	0.30
NRPS1	947	5	130(D→N), 270(T→A), 351(T→A), 450(L→M), 510(F→M)	0.53
NRPS2	2,394	16	70(K→N), 190(F→G), 329(L→I), 391(C→S), 492(S→T), 550(D→E), 557(S→C), 630(Y→N), 829(F→W), 910(K→M), 1090(S→M), 1290(Q→K), 2069(R→S), 2211(G→D), 2290(K→N), 2397(E→S)	0.67
NRPS3	2,387	15	110(K→N), 170(Y→C), 312(F→Y), 371(N→S), 393(N→I), 431(Q→H), 669(E→G), 788(G→V), 1292(K→N), 1373(E→G), 1514(Q→L), 1670(L→W), 2172(P→Q), 2386(E→S), 2387(I→E)	0.63

### Variation in Gene Expression Levels of Lrp and NRPS-PKS Between Two *X. hominickii* Strains

**FIGURE 9:**
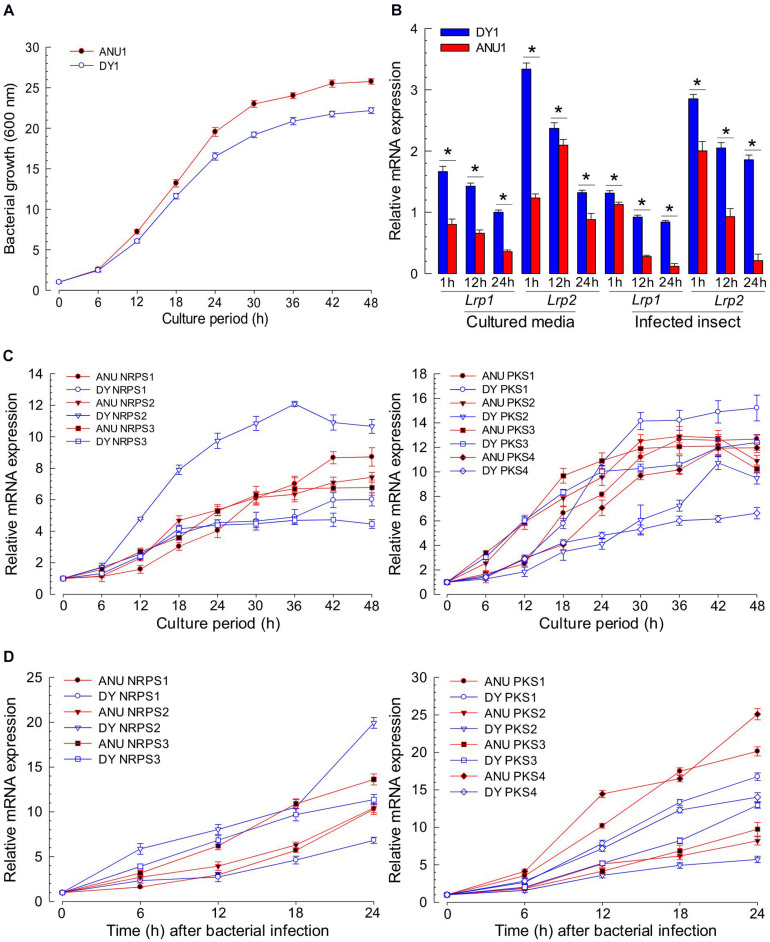
Comparative analysis of NRPS-PKS gene expression between two strains of *X. hominickii*. **(A)** Their growth curves at 28°C in TSB broth. **(B)** Temporal expression levels of two Lrp genes during bacterial growth. Asterisks above standard error bars indicate significant differences between means of two strains at each time pint at Type I error = 0.05 (LSD test). **(C)** Comparative expression analysis of three NRPS (left panel) and four PKS (right panel) genes between the two strains during bacterial growth in TSB broth. **(D)** Comparative expression analysis of three NRPS (left panel) and four PKS (right panel) genes between two strains during bacterial growth in *S. exigua*. Each L5 larva was infected with 10^2^ cfu of *X. hominickii*. Each treatment was independently replicated three times.

To clarify the difference in the production of secondary metabolites, Lrp and NRPS-PKS genes were assessed for their expression levels during bacterial growth (Figure 9). In our culture condition, both strains grew well, reaching to stationary phase at 30 h after the initial inoculation (Figure 9A). In expression analyses of two *Lrp* genes, DY1 strain exhibited higher expression levels than ANU1 strain at all bacterial growth phases (Figure 9B). Regarding *NRPS* expression levels, the two strains exhibited significantly (*F* = 3,491.5; *df* = 1, 108; *P* < 0.0001) different patterns (Figure 9C). For PKS expression levels, the two strains also showed significantly (*F* = 10.7; *df* = 1, 144; *P* < 0.0001) different patterns. NRPS-PKS gene expression patterns in *S. exigua* infected with *X. hominickii* were also investigated (Figure 9D). For *NRPS* expression levels, the two bacterial strains exhibited significantly (*F* = 154.7; *df* = 1, 60; *P* < 0.0001) different patterns, although they were not significantly (*F* = 3.9; *df* = 1, 7; *P* = 0.0894) different at an initial stage (6 h after infection). For *PKS* expression levels, the two bacterial strains also exhibited significantly (*F* = 1,428.7; *df* = 1, 80; *P* < 0.0001) different patterns.

## Discussion

This study reports that two strains of *X. hominickii* have different virulence. These two strains (ANU1 and DY1) were identical in biochemical and molecular diagnosis characters. However, they showed marked difference in their compositions of secondary metabolites. They also exhibited genetic difference for genes associated with the production of secondary metabolites.

These two strains of *X. hominickii* were different in virulence against two lepidopteran insects (*S. exigua* and *M. vitrata*) and one coleopteran insect (*T. molitor*), with ANU1 being more potent than DY1. Based on LD_50_ values, ANU1 strain was about twice more potent than DY1 against *S. exigua* (228.1 vs. 502.1 cfu/larva) and *M. vitrata* (152.3 vs. 322.6 cfu/larva). Their difference in virulence was greater against *T. molitor* (666.8 vs. 3,573.3 cfu/larva), with ANU1 strain being five times more potent than DY1. Different virulence of bacteria species has been reported for *X. nematophila* (Hasan et al., 2019) and *P. temperata temperata* (Ahmed and Kim, 2018). Septicemia induced by bacterial injection has been reported as a main pathogenicity to kill target insects (Park and Kim, 2000; Cho and Kim, 2004). Immunosuppression might have been accomplished after the induction of septicemia which is usually induced by entomopathogenic bacteria via inhibition of eicosanoid biosynthesis with their secondary metabolites (Seo et al., 2012). This is because eicosanoids can mediate both cellular and humoral immune responses in insects (Kim et al., 2018). The difference in virulence can also be explained by their genetic factors. For example, epigenetic control of bacterial genome by DNA methylation can alter mobility and insecticidal activity of both *Xenorhabdus* and *Photorhabdus* (Payelleville et al., 2017). Structural change of bacterial outer membrane contributes to the pathogenicity of *X. nematophila*. Such change is induced by differential expression of a virulence modulation gene called opaB (Park et al., 2007). These findings suggest that the difference in virulence between the two *X. hominickii* strains might be due to genetic or epigenetic variations in virulence-associated genes, which in turn can modulate the production of secondary metabolites including eicosanoid biosynthesis inhibitors.

The two strains of *X. hominickii* produced and released secondary metabolites into their culture broth. However, their compositions were different. Based on GC-MS analysis of their organic extracts, 121 secondary metabolites were produced by the two strains, including indole derivatives, cyclopeptides, pyrollopyrazines, piperazines, and phenylethyl amines. ANU101 strain produced more secondary metabolites than DY1. Especially, ANU1 strain produced all five kinds of indole derivatives while DY1 strain only produced indole. Cyclopeptides, pyrollopyrazines, piperazines, and phenylethyl amines are known products of NRPS-PKS (Shi and Bode, 2018). These NRPS-PKS products were also different between the two strains. Whole genome sequencing of *Xenorhabdus* and *Photorhabdus* has revealed their biosynthesis potential for secondary metabolites, with 7.5% of *X. nematophila* genome containing biosynthesis genes associated with secondary metabolites (Chaston et al., 2011). Therefore, although several secondary metabolites from entomopathogenic bacteria are identical (Tobias et al., 2018), a number of unidentified secondary metabolites might be potentially produced based on their genetic background.

Indole and its derivatives produced by *X. hominickii* showed immunosuppressive and insecticidal activities in the present study. Indole derivatives produced by *X. nematophila* have also been reported to exhibit potential activities to keep a monoxenic condition in target insect cadaver by accumulating guanosine-3′,5′-bis-pyrophosphate, a regulatory nucleotide, in susceptible bacteria (Sundar and Chang, 1993). Indole and four derivatives have been identified from *X. bovienii* (Chen et al., 1996). Our current study identified four novel indole derivatives: tryptophol, indole acetyl hydrazine, methyl phenyl indole, and methyl tryptophan. Tryptophol and indole acetyl hydrazine are similar to xenocyloins produced by *X. bovienii* (Shi and Bode, 2018). Except for methyl tryptophan that was untested, indole and three derivatives inhibited the activity of insect PLA_2_, suppressed the cellular immune response, and exhibited insecticidal activities after oral administration. Among four compounds tested, methyl phenyl indole was the most potent one. It was only detected in the ANU1 strain, but not in DY1. Other indole derivatives were also detected in the ANU1 strain, but not in DY1. Such differences in these potent secondary metabolites might explain the different virulence between the two *X. hominickii* strains.

GC-MS analysis of bacterial metabolites revealed 121 secondary metabolites. They might have been synthesized by catalytic activities of gene products of NRPS-PKS. Three NRPS and four PKS loci were predicted from the genome of *X. hominickii*. Their expressions were confirmed by RT-PCR. These two are multi-enzymes with assembly lines that can catalyze the sequential condensation of simple building blocks of acyl-CoA thioesters and amino acids, respectively (Weissman and Leadlay, 2005; Walsh, 2008). Chain extension is accomplished by successive modules of enzymes. Thus, genetic organization is colinear with the sequence of biosynthetic transformations. PKS modules can minimally incorporate acyl transferase and ketosynthase domains required for the selection of a specific building block and its incorporation via a thioclaisen-like condensation into a growing chain. The resulting intermediate can undergo redox adjustment at variable extent depending on the specific complement of reductive domains (ketoreductase, dehydratase, and enoyl reductase) present within the module. Throughout biosynthesis, chain extension intermediates are tethered to these multi-enzymes in thioester linkage to phosphopantetheine prosthetic group of integral acyl carrier protein of each module. This architecture allows chains to be shuttled efficiently between various active sites. Analogous core functions of NRPS include condensation (or heterocyclization) and peptidyl carrier protein domains, whereas optional modifying enzymes may comprise epimerase, methyl transferase, and oxidase functions (Walsh et al., 2001). Chain termination in both systems is typically due to an integral thioesterase activity (Kopp and Marahiel, 2007). The existence of hybrid PKS-NRPS multi-enzymes reflects a shared biosynthetic logic of the two systems. The two isolates of *X. hominickii* were different in sequences of these loci. These two strains shared over 99% sequence identities for all seven loci. However, a number of point mutations were detected between the two strains. With respect to amino acid sequences, these two strains showed genetic variations ranging from 0.12% in PKS to 0.67% in NRPS. There were significant variations in all NRPS-PKS genes between ANU1 and DY1 strains. Furthermore, we detected significant variations in a global transcription factor, Lrp, between ANU1 and DY1 strains. Lrp is a virulence modulator. Low *Lrp* expression is associated with a virulent phenotype and suppression of antimicrobial peptides (AMP) in *Manduca sexta* while a high *Lrp* level can reduce virulence and AMP expression (Casanova-Torres et al., 2017). Thus, specific *Lrp* expression level may influence expression levels of *NRPS-PKS*, leading to the production of secondary metabolites. All tested NRPS-PKS-associated secondary metabolites suppressed immune responses and exhibited insecticidal activities in the present study.

From these results, the differential virulence of the two isolates of *X. hominickii* can be explained by variations in their production of secondary metabolites modulated by differential *Lrp* expression levels. Especially, indole derivatives were predominantly produced only in the ANU1 strain. In addition, two potent piperazines presumably produced by NRPS-PKS were only detected in the ANU1 strain. Such differential production of secondary metabolites was supported by genetic variations in NRPS-PKS loci between these two strains.

## Data Availability Statement

The datasets presented in this study can be found in online repositories. The names of the repository/repositories and accession number(s) can be found in the article/Supplementary Material.

## Author Contributions

YK designed the work. MM analyzed secondary metabolites. MR assessed nematode and bacterial pathogenicity. D-YC isolated *X. hominickii* DY1. MH identified the nematode. MA sequenced NRPS-PKS loci of *X. hominickii* DY1. H-SY synthesized indole derivatives. YK and MM wrote the manuscript. All authors contributed to the article and approved the submitted version.

## Conflict of Interest

The authors declare that the research was conducted in the absence of any commercial or financial relationships that could be construed as a potential conflict of interest.

## Footnotes

1 http://www.ncbi.nlm.nih.gov

2 http://nistmassspeclibrary.com

3 http://nrps.igs.umaryland.edu/

4 https://pfam.xfam.org
