# Supplementary material for: Variations of Indole Metabolites and NRPS-PKS Loci in Two Different Virulent Strains of Xenorhabdus hominickii
Source: Front Microbiol. 2020 Nov 24;11:583594. doi: 10.3389/fmicb.2020.583594 (PMC7732475; doi:10.3389/fmicb.2020.583594)
Supplement: Supplementary file 1 [file Data_Sheet_1.PDF]

SUPPLEMENTARY MATERIAL

TABLE S1 | Primers used in this study for amplification of PKS and NRPS from *X. hominickii* DY1

TABLE S2 | Identification of an entomopathogenic nematode isolate with morphological characters of infected juveniles

TABLE S3 | Genus identification of an entomopathogenic bacteria with physiological and biochemical characters

TABLE S4 | Species identification of an entomopathogenic bacterium with carbon usage characters assessed with Biolog microbial identification using Gen III plate

TABLE S5 | GC-MS analysis of organic extracts of two *X. hominickii* strains organic solvents used for extraction: *X. hominickii* ANU1 ('ANU') and *X. hominickii* DY1 ('DY').

FIGURE S1. Identification of a bacterial isolate from *S. monticolum* using 16S rRNA sequence (GenBank accession number: MH997838.1). (**A**) 16S rRNA sequences of three blue colonies (#1-3) on NBTA culture medium in three independent colonies. DNA sequence and Blast search are shown in each colony. (**B**) Phylogeny analysis with bacterial species of *Xenorhabdus* and *Photorhabdus*. The tree was constructed as described in Figure 2. GenBank accession numbers of ITS/rRNA sequences followed their own species names.

FIGURE S2. Variation of secondary metabolites in the cultured broth of two *X. hominickii* strains ('ANU101' and 'DY1'). Metabolites were separated on TLC. (A) A pictorial representation of TLC analysis (B) Summary of TLC analysis based on retention index (RI). For TLC, composition of chloroform, methanol and acetic acid (7.5:2.0:0.5, v/v) was used as eluent.

FIGURE S3. Sequence alignment of NRPS-PKS of two *X. hominickii* strains ('ANU101' and 'DY1'). Primers were designed from *X. hominickii* ANU101 nucleotide sequence. NRPS-PKS sequences were read in a chromosomal walking manner with sequential primers for each specific region. The resulting PCR product was bidirectionally sequenced.

FIGURE S4. Alignment of two Lrp genes encoded in *X. hominickii* ANU1 in nucleotide (A) and amino acid (B) sequences. Alignment was performed using ClustalW multiple sequence alignment program (https://www.ebi.ac.uk). Asterisks indicate the conserved residues. Semicolons indicate the similar type of amino acids in polarity.

FIGURE S5. A phylogeny analysis of *X. hominickii Lrp1* and Lrp/AsnC (*Lrp2*) with other *Xenorhabdus* and *Photorhabdus* spp. The tree was constructed with the Neighbor-joining method using MEGA6. Bootstrapping values on branches were obtained with 1,000 repetitions. GenBank accession numbers followed their own species names.

TABLE S1 | Primers used in this study for amplification of PKS and NRPS from *X. hominickii* DY1

Genes	Primer Sequence	Annealing temperature (°C)	Amplicon size (bp)
PKS1-1	5′-TCC AAC GGT GGC CGA TAA AA-3′<br>5′-TGC TCA AGG TGC TGA CGA AT-3′	59.0 & 59.0	1121
PKS1-2	5′-TGT TGC TTG TGG CCG AAT TG-3′<br>5′-GAT ACC GGC AAG ATA CGG CA-3′	59.0 & 59.0	1070
PKS1-3	5′-TGC CGT ATC TTG CCG GTA TC-3′<br>5′-TCA CTG ACG ATT GCG ACC AA-3′	59.0 & 59.0	1254
PKS1-4	5′-AGT TGG TCG CAA TCG TCA GT-3′<br>5′-CCG CCA GCG TTT TCT TTT CA-3′	59.0 & 59.0	1243
PKS1-5	5′-CGG GCG ATT TAC CTG CAT TG-3′<br>5′-CGT AAT TTG GCA TGA GCG GG-3′	59.0 & 59.0	1060
PKS2-1	5′-CTG TGC ATC GGC ACA ATA CG-3′<br>5′-CGT AAA GCG TAA ATG CCG GG-3′	59.0 & 59.0	1156
PKS2-2	5′-CCC GGC ATT TAC GCT TTA CG-3′<br>5′-CGT GGT ATT GCA TTC TGC CG-3′	59.0 & 59.0	1245
PKS2-3	5′-CGG CAG AAT GCA ATA CCA CG-3′<br>5′-ACC ACC GCG ATA AAG GGT TT-3′	59.0 & 59.0	1090
PKS3-1	5′-TAG TTC CAG CAG CAG TTA TTT-3′<br>5′-TTC CTG CTC CAT CGT TAT TG-3′	54.1 & 54.6	1176
PKS3-2	5′-CGA TGG AGC AGG AAA GAT AG-3′<br>5′-AAC TGA CGA ACC CAG TAA TC-3′	54.5 & 54.1	1786
PKS3-3	5′-CGTTCAGGCAGGCATTTA-3′<br>5′-GATGAAACAGGGCTAAGTAGAG-3′	54.4 & 55.0	1521
PKS3-4	5′-ACG TTC GTC CAC CAT TTC-3′<br>5′-GGT TCT CCT GCG GTT TAT T-3′	54.4 & 54.2	1287
PKS4	5′-ATGCACTGCCGGATGAAA-3′<br>5′-TCTAGCTCCTTCACCTTACCA -3′	62.0 & 62.0	761
NRPS1-1	5′-TACTTCATCAACACCTTGGG-3′<br>5′-TATCCAGATTAGTGAGGGCA-3′	54.0 & 54.0	1116
NRPS1-2	5′-CCCCTTACAGAGATAAACCG-3′<br>5′-TGGATTCGGTGAGCAAATAA-3′	54.0 & 54.1	1804
NRPS2-1	5′-CTTGTCATCGGAACTTATG-3′<br>5′-CACGGCATCTAAACTATTC-3′	50.1 & 50.2	1957
NRPS2-2	5′-CAATAACAGGAGCATCAG-3′<br>5′-GGCTGTGTCAAGTTTAG-3′	49.1 & 48.8	1949
NRPS2-3	5′-GATAGGCCACTTCAAATC-3′<br>5′-CGGAGGATAGCGATAAT-3′	49.1 & 48.7	1364
NRPS2-4	5′-CGATCCCAATTCTCATTC-3′<br>5′-GCTGAACTTGCTTTATCT-3′	49.2 & 48.9	1992
NRPS2-5	5′-CGCCAATTCCAACTATC-3′<br>5′-CGGACGACCTACATTAT-3′	49.1 & 48.9	458
NRPS3-1	5′-CAAGCAGAAGGTTTGAG-3′<br>5′-GACTTTGACACCACTATC-3′	49.1 & 48.7	1449
NRPS3-2	5′-TGACGATCAACCTACTC-3′<br>5′-CTGCTCTCTATCCCAAA-3′	48.8 & 48.5	1075
NRPS3-3	5′-ATGGTACCCGACAATC-3′<br>5′-GCAGGTGATGGAAGTA-3′	48.4 & 48.3	1339
NRPS3-4	5′-CATAGTCAGCAGAGTAAAG-3′	49.0 & 48.4	1909

	5′-CTCCACCAGAAGTAAGT-3′		
NRPS3-5	5′-CCACTCACACAAAGAAG-3′<br>5′-GCCAGATCACACAATAC-3′	48.7 & 48.6	1599
NRPS3-6	5′-GCCAGCCAGAATTAAC-3′<br>5′-CCGAAGCCAACAATAC-3′	48.3 & 48.4	1578

TABLE S2 | Identification of an entomopathogenic nematode isolate with morphological characters of infected juveniles

Characters	Isolate	*Steinernema monticolum*[1]
Total body length (µm)	679.0 ± 6.1	706 (612-821)
Maximum body width (µm)	30.7 ± 3.3	37 (32-46)
Head to excretory pore length (µm)	97.0 ± 4.5	58 (54-62)
Tail length (µm)	71.6 ± 10.0	77 (71-95)
Esophagus length (µm)	128.2 ± 8.5	124 (120-136)

[1] Identifying characters of *Steinernema monticolum* according to Stock et al. (1997)

TABLE S3 | Genus identification of an entomopathogenic bacteria with physiological and biochemical characters

Characters	Isolate	*Xenorhabdus*	*Photorhabdus*
Nematode host	*Steinernema monticolum*	*Steinernema* spp.	*Heterorhabditis* spp.
Gram stain	-	-	-
Oxidase production	-	-	-
Catalase	-	-	±
Bioluminescence	-	-	±
Cadaver color	Yellow	Yellow	Brick red
Mobility	+	+	+

TABLE S4 | Species identification of an entomopathogenic bacterium with carbon usage characters assessed with Biolog microbial identification using Gen III plate

Carbon source	Responses[1] of *Xenorhabdus* spp.[2]			
	Isolate	Xh	Xb	Xn
D-Aspartic Acid, D-Trehalose, N-Acetyl-D-Glucosamine, α-D-Glucose, D-Mannose, Inosine, Glycerol, D-Glucose-6-PO_4, Troleandomycin, Gelatin, Glycyl-L-Proline, L-Arginine, L-Glutamic Acid, L-Pyroglutamic Acid, L-Serine, Vancomycin, Tetrazolium Violet, Tetrazolium Blue, Acetic Acid	+	+	+	+
D-Maltose, 1% Sodium Lactate, Rifamycin SV, D-Saccharic Acid, D-Malic Acid	+	+	+	-
D-Arabitol, myo-Inositol, D-Serine, L-Aspartic Acid, Lincomycin, L-Lactic Acid, L-Malic Acid, Potassium Tellurite, Tween 40, α-Keto-Butyric Acid, Acetoacetic Acid	+	+	-	+
D-Cellobiose, D-Turanose, β-Methyl-D-Glucoside, N-Acetyl-β-D-Mannosamine, N-Acetyl-D-Galactosamine, 3-Methyl Glucose, L-Alanine, L-Histidine, Pectin, D-Gluconic Acid, Mucic Acid, Aztreonam	+	+	-	-
Dextrin, D-Fructose-6-PO_4, L-Galactonic Acid Lactone, p-Hydroxyphenylacetic Acid, Sodium Butyrate	-	-	+	+
Lithium Chloride	-	-	+	-
L-Rhamnose, Methyl Pyruvate, α-Hydroxy-Butyric Acid, Propionic Acid, Formic Acid	-	-	-	+
D-Serine	-	+	+	-
D-Fructose	+	-	+	+
Similarity (%)	100	96.7	50.0	55.0

[1] '-' for < 34% and '+' for > 34%
[2] 'Xh' for *Xenorhabdus hominickii*, 'Xb' for *X. bovienii*, and 'Xn' for *X. nematophila*

TABLE S5 | GC-MS analysis of organic extracts of two *X. hominickii* strains organic solvents used for extraction: *X. hominickii* ANU1 ('ANU') and *X. hominickii* DY1 ('DY').

SN	RT	Chemical Name	Quality	Extraction				MW	strains	
				HEX	EAX	CX	BX		ANU	DY
1	2.394	N-butyl-1-Butanamine	91	+	+	+	+	129.152	●	●
2	14.419	Cetene	99	+				224.25	●	●
3	18.758	1-Octadecene	99	+				252.282	●	●
4	22.719	1-Nonadecene	99	+				266.297	●	●
5	31.952	Bis(2-ethylhexyl) phthalate	99	+	+	+		390.277	●	●
6	9.761	E-2-tetradecene	98	+				196.219	●	
7	9.956	Tetradecane	98	+		+		198.235	●	●
8	11.184	Phthalimide	98	+		+	+	147.032	●	●
9	14.602	Hexadecane	98	+	+	+	+	226.266	●	●
10	18.911	Octadecane	98	+		+		254.297	●	●
11	22.843	Eicosane	98	+		+		282.329	●	●
12	9.72	1-Tetradecene	98	+				196.219		●
13	7.418	Indole	97	+	+			117.058	●	●
14	12.306	Pentadecane	97	+		+		212.25	●	●
15	12.506	2,4-Bis(1,1-dimethylethyl)-phenol	97	+				206.167	●	●
16	16.804	Heptadecane	97	+	+	+		240.282	●	●
17	17.524	Hexahydro-Pyrrolo[1,2-a]pyrazine-1,4-dione	97			+	+	154.074	●	●
18	29.178	Hexahydro-3-(phenylmethyl)-pyrrolo[1,2-a]pyrazine-1,4-dione	97		+	+	+	244.121	●	●
19	7.01	4-Methyl-5-thiazoleethanol	96		+	+		143.04	●	
20	20.919	Nonadecane	96	+				268.313	●	●
21	21.633	3,5-Bis(1,1-dimethylethyl)-4-hydroxy-benzenepropanoic acid methyl ester	96	+				292.204	●	
22	3.031	Benzyl alcohol	95		+			108.058	●	
23	4.041	Phenylethyl Alcohol	95		+			122.073	●	
24	7.618	N,N-dibutyl-Formamide	95			+		157.147	●	
25	20.824	Hexadecanenitrile	95			+		237.246	●	
26	21.615	Hexahydro-3-(2-methylpropyl)-pyrrolo[1,2-a]pyrazine-1,4-dione,	95		+	+	+	210.137	●	●
27	24.213	Oleanitrile	95			+		263.261	●	
28	25.83	N-[2-(1H-indol-3-yl)ethyl]-Acetamide	95		+			202.111		●
29	11.456	o-Cyanobenzoic acid	94	+				147.032	●	
30	11.928	Hexathiane	94	+	+			191.832	●	
31	22.932	Cyclic octaatomic sulfur	94	+	+	+	+	255.777	●	

32	29.75	Docosane	94	+		+		310.36	●	
33	15.688	7,9-Dimethyl-1,4-dioxa-7,9-diazacycloundecane-8-thione	93	+				218.109	●	
34	24.679	1-Chloro-nonadecane	93	+				302.274	●	
35	27.235	3-Benzyl-6-isopropyl-2,5-piperazinedione	93		+			246.137	●	●
36	21.013	Diphenyl sulfone	93	+	+			218.04		●
37	19.773	2-Butyl-5-hexyloctahydro-1H-indene	92	+				264.282	●	
38	24.679	Nonadecanenitrile	92			+		279.293	●	
39	10.375	4-Hydroxy-benzeneethanol	91		+	+		138.068	●	
40	32.737	9-Hexacosene	91	+				364.407	●	
41	2.618	Butyl ester butanoic acid	90				+	144.115	●	
42	4.478	2,3-Dihydro-3,5-dihydroxy-6-methyl-4H-pyran-4-one	90		+			144.042	●	●
43	12.335	N-(2-Phenylethyl)- Acetamide	90		+	+		163.1	●	●
44	21.131	2-Dodecen-1-yl(-)succinic anhydride	90	+				266.188	●	
45	25.588	3-Methyl-6-(phenylmethyl)-2,5-piperazinedione	90			+		218.106	●	
46	25.724	3-(Phenylmethyl)-2,5-piperazinedione	90		+	+		204.09	●	
47	28.977	1-Eicosene	90	+				280.313	●	
48	4.112	Diacetate-1,3-butanediol	90		+			174.089		●
49	9.283	Propanoic acid, 2-methyl-, 3-hydroxy-2,4,4-trimethylpentyl ester	86			+		216.173	●	
50	13.935	Tridecane	86	+				184.219	●	
51	21.391	Hexadecanoic acid, methyl ester	86	+				270.256	●	●
52	3.018	2-Ethyl-1-hexanol	85				+	130.136	●	
53	3.864	Benzeneethanamine	83			+	+	121.089	●	●
54	16.94	2,6,10-Trimethyl-Dodecane	83	+				212.25	●	
55	14.962	Tryptophol	81	+	+			161.084	●	
56	14.974	1H-Indole-3-acetic acid, hydrazide	81		+			189.09	●	
57	3.828	2-Propenylidene-cyclobutene	80			+		92.063		●
58	6.638	1,1-Diisobutoxy-isobutane	78				+	202.193	●	
59	2.565	1,2-Ethanediol, diacetate	78	+		+		146.058		●
60	25.488	L-Tryptophan, methyl ester	74			+		218.106	●	
61	10.452	N-(3-Methylbutylidene)-benzeneethanamine,	72				+	189.152	●	●
62	24.466	E-8-Methyl-9-tetradecen-1-ol acetate	70			+		268.24	●	
63	11.367	(2-Isothiocyanatoethyl)-benzene	64				+	163.046	●	●
64	18.321	4,5-Dihydro-3-methyl-1-propyl-1H-pyrazole	64					126.116	●	
65	20.777	11,13-Dimethyl-12-tetradecen-1-ol acetate	64	+				282.256	●	
66	22.194	Tetradecyl- oxirane	64	+				240.245	●	
67	25.966	E-11-Methyl-12-tetradecen-1-ol acetate	64	+				268.24	●	

68	26.149	d-Leucyl-d-leucine, trimethylsilyl ester	64		+		+	316.218		●
69	31.604	5-Methyl-2-phenyl-1H-Indole	64		+	+		207.105	●	
70	17.878	4-Mercacptophenol	64		+			126.014		●
71	15.606	1-bromo-octadecane	60	+				332.208	●	
72	17.383	2-(Methylthio)-1H-benzimidazole	60	+				164.041	●	
73	22.276	2-Mercaptobenzothiazole	60			+		166.986	●	
74	24.095	9-Borabicyclo[3.3.1]9-(3-methoxycyclohexyl)oxy-nonane	60			+		250.21	●	
75	26.196	Nadolol	60				+	309.194	●	
76	8.799	Propanoic acid, 2-methyl-, 2,2-dimethyl-1-(2-hydroxy-1-methylethyl)propyl ester	59			+		216.173	●	
77	16.166	2-Methyl-Z-4-tetradecene	59	+				210.235	●	
78	17.223	(3S,6S)-3-Butyl-6-methylpiperazine-2,5-dione	59			+	+	184.121	●	●
79	19.974	6-methyl-8-(2,6,6-trimethyl-1-cyclohexen-1-yl)-5-Octen-2-one	59	+				262.23	●	
80	26.669	2-Hydroxy-3,5,5-trimethyl-cyclohex-2-enone	59		+	+	+	154.099	●	●
81	28.752	Cyclo-(l-leucyl-l-phenylalanyl)	59		+	+		260.152	●	●
82	19.301	Dicyclohexyldisulphide	58	+				230.116	●	
83	21.781	3-Dodecyl-2,5-furandione	58	+				266.188	●	
84	26.356	4-(Diethylamino)- 2-butanone,	58		+			143.131	●	
85	16.125	2-[(1-methylethyl)thio]- Phenol	56		+			168.061	●	
86	20.128	4-(4-Ethylcyclohexyl)-1-pentyl-cyclohexene	56	+				262.266	●	
87	7.412	4-Methyl-2-(1-methylethyl)-Thiazole	55				+	141.061	●	
88	13.758	1,5-Dimethyl-7-oxabicyclo[4.1.0]heptane	55					126.104	●	
89	17.012	3-Ethoxy-4-methoxyphenol	55		+			168.079	●	
90	17.611	2-Chlorophenoxyacetic acid hydrazide	55		+			200.035	●	
91	19.03	14-Methyl- (Z)- 8-Hexadecenal,	55	+				252.245	●	
92	7.205	4-Octanone	53			+		128.12	●	
93	13.764	Oxalic acid, allyl dodecyl ester	53	+				298.214	●	
94	18.463	1,2-Bis-(2-diisopropylaminoethyl) ethane	53		+			228.257	●	
95	21.851	5-Isopropylidene-3,3-dimethyl-dihydrofuran-2-one	53			+		154.099	●	●
96	34.839	Decamethyl- tetrasiloxane	53	+				310.127	●	
97	17.312	1,3-dimethyl-3,4,5,6-tetrahydro-2(1H)-pyrimidinone	53		+			128.095		●
98	16.686	3-Phenoxy-Phenol	52	+				186.068	●	
99	16.869	Diisopropylcyanamide	50		+			126.116	●	
100	27.182	Diethyldithiophosphinic acid	50			+		154.004	●	
101	5.292	4,4-(Ethylenedioxy)-2-pentanone	50		+			144.079		●

102	17.967	Cyclo-(glycyl-l-leucyl)	50		+			170.106		●
103	16.633	2,3-Dimethyl-1,4-naphthalenedione	50	+				186.068		●
104	17.371	1-Methyl hydrouracil -	49			+		128.059		●
105	19.508	3,5-Dimethoxy-phenol	43				+	154.063	●	
106	2.577	2-Methyl-propanoic acid, 1-methylethyl ester	43				+	130.099		●
107	19.596	2-(Dimethylamino)-1-phenyl-4-octanone	43		+			247.194		●
108	16.29	4H-1,2,4-Triazol-4-amine	38		+		+	84.044		●
109	31.326	4-(3-Phenylpropyl)benzoic acid, ethyl ester	38			+		268.146		●
110	11.928	Benzenebutanoic acid, ethyl ester	37		+	+		192.115		●
111	16.615	3-Pyrrolidin-2-yl-propionic acid	37		+	+		143.095		●
112	12.571	4,8-Dimethyl-2(1H)-quinolinone,	35			+		173.084	●	
113	12.058	2-(2,4,6-Trimethylphenyl)-Thiazole	35	+				203.077		●
114	30.205	4-(Phenylmethoxy)-1,2,5-oxadiazol-3-amine	18			+		191.069		●
115	17.973	Heptadecanal	15	+				254.261		●
116	21.928	1,2-Benzenedicarboxylic acid, bis(2-methylpropyl) ester	90	+				278.152		●
117	22.56	2,6-Dimethyl-4-nitro-3-phenyl-cyclohexanone	30	+				247.121		●
118	25.287	4,8,12,16,20,24,28-Heptamethyl-1,5,9,13,17,21,25-heptaoxa-cyclooctacosane-2,6,10,14,18,22,26-heptaone	45	+				602.257		●
119	26.781	3-Methyl-3-(4,8-dimethylnona-3,7-dienyl)-4-methylene-1,3-dioxolan-2-one	55	+				264.173		●
120	28.929	9-Octadecenamide, (Z)-	96	+				281.272		●
121	31.917	Di-n-octyl phthalate	91	+				390.277		●
∑				57	40	45	21		93	57

(A)

[Colony #1]

CACTGGAAACGGTGGCTAATACCGCATGACCTCTGAGGAGCAAAGTGGGGGACCTTCGGGCCTCACGCCATCGGA
TGAACCCAGATGGGATTAGCTAGTAGGTGAGGTAACGGCTCACCTAGGCGACGATCCCTAGCTGGTCTGAGAGGA
TGACCAGCCACACTGGGACTGAGACACGGCCCAGACTCCTACGGGAGGCAGCAGTGGGGAATATTGCACAATGGG
CGCAAGCCTGATGCAGCCATGCCGCGTGTATGAAGAAGGCCTTCGGGTTGTAAAGTACTTTCAGCGGGGAGGAAG
GCGTGAAGTTTAACAGACTTTACGATTGACGTTACCCGCAGAAGAAGCACCGGCTAACTCCGTGCCAGCAGCCGC
GGTAATACGGAGGGTGCAAGCGTTAATCGGAATTACTGGGCGTAAAGCGCACGCAGGCGGTCAATTAAGTTAGAT
GTGAAATCCCCGGGCTTAACCTGGGAACTGCATCTAAAACTGATTGGCTAGAGTCTCGTAGAGGGGGGTAGAATT
CCACGTGTAGCGGTGAAATGCGTAGAGATGTGGAGGAATACCGGTGGCGAAGGCGGCCCCCTGGACGAAGACTGA
CGCTCAGGTGCGAAAGCGTGGGGAGCAAACAGGATTAGATACCCTGGTAGTCCACGCTGTAAACGATGTCGATTT
GGAGGTTGTGCCCTTGAGGCGTGGCTTCCGGAGCTAACGCGTTAAATCGACCGCCTGGGGAGTACGGCCGCAAGG
TTAAAACTCAAATGAATTGACGGGGGCCCGCACAAGCGGTGGAGCATGTGGTTTAATTCGATGCAACGCGAAGAA
CCTTACCTACTCTTGACATCCAGCGAATCCTTTAGAGATAGAGGAGTGCCTTCGGGAACGCTGAGACAGGTGCTG
CATGGCTGTCGTCAGCTCGTGTTGTGAAATGTTGGGTTAAGTCCCGCAACGAGCGCAACCCTTATCCTTTGTTGC
CAGCACGTAATGGTGGGAACTCAAGGGAGACTGCCGGTGATAAACCGGAGGAAGGTGGGGATGACGTCAAGTCAT
CATGGCCCTTACGAGTAGGGCTACACACGTGCTACAATGGCAGATACAAAGAGAAGCGACCTCGCGAGAGCAAGC
GGAACTCATAAAGTCTGTCGTAGTCCGGATTGGAGTCTGCAACTCGACTCCATGAAGTCGGAATCGCTAGTAATC
GTAGATCAGAATGCTACGGTGAATACGTTCCCGGGCCTTGTACACACCGCCCGTCACACCATGGGAGTGGGTTGC
AAAAGAAGTAGGTAGCTTAACCTTCGGGAGGGCGCTTACCACTTTGTGATTCATGACTGGGGTGAAGTCGTAACA
AGGTAGCCGT

Accession No.	Species	Gene	Max scores	E-value	Identity (%)
CP016176.1	*X. hominickii* strain ANU1	16S rRNA	2512	0.0	100
AB507814.1	*X. hominickii* strain HkNk135		2507	0.0	99.3
FJ515811.1	*X. hominickii* strain Sm1		2507	0..0	99.3

[Colony #2]

GAGCTCGGATCCACTAGTAACGGCCGCCAGTGTGCTGGAATTCGCCCTTTACGGCTACCTTGTTACGACTTCACC
CCAGTCATGAATCACAAAGTGGTAAGCGCCCTCCCGAAGGTTAAGCTACCTACTTCTTTTGCAACCCACTCCCAT
GGTGTGACGGGCGGTGTGTACAAGGCCCGGGAACGTATTCACCGTAGCATTCTGATCTACGATTACTAGCGATTC
CGACTTCATGGAGTCGAGTTGCAGACTCCAATCCGGACTACGACAGACTTTATGAGTTCCGCTTGCTCTCGCGAG
GTCGCTTCTCTTTGTATCTGCCATTGTAGCACGTGTGTAGCCCTACTCGTAAGGGCCATGATGACTTGACGTCAT
CCCCACCTTCCTCCGGTTTATCACCGGCAGTCTCCCTTGAGTTCCCACCATTACGTGCTGGCAACAAAGGATAAG
GGTTGCGCTCGTTGCGGGACTTAACCCAACATTTCACAACACGAGCTGACGACAGCCATGCAGCACCTGTCTCAG
CGTTCCCGAAGGCACTCCTCTATCTCTATAGGATTCGCTGGATGTCAAGAGTAGGAAAGGGTCTTCGCGTTGCAT
CAAATTAAACCACATGCTCCACCGCTTGTGCGGGCCCCCGTCAATTCATTTGAGTTTTAACCTTGCGGCCGTACT
CCCCAGGCGGTCGATTTAACGCGTTAGCTCCGGAAGCCACGCCTCAAGGGCACAACCTCCAAATCGACATCGTTT
ACAGCGTGGACTACCAGGGTATCTAATCCTGTTTGCTCCCCACGCTTTCGCACCTGAGCGTCAGTCTTCGTCCAG
GGGGCCGCCTTCGCCACCGGTATTCCTCCACATCTCTACGCATTTCACCGCTACACGTGGAATTCTACCCCCCTC
TACGAGACTCTAGCCAATCAGTTTTAGATGCAGTTCCCAGGTTAAGCCCGGGGATTTCACATCTAACTTAATTGA
CCGCCTGCGTGCGCTTTACGCCCAGTAATTCCGATTAACGCTTGCACCCTCCGTATTACCGCGGCTGCTGGCACG
GAGTTAGCCGGTGCTTCTTCTGCGGGTAACGTCAATCGTAAAGTCTGTTAAACTTCACGCCTTCCTCCCCGCTGA
AAGTACTTTACAACCCGAAGGCCTTCTTCATACACGCGGCATGGCTGCATCAGGCTTGCGCCCATTGTGCAATAT
TCCCCACTGCTGCCTCCCGTAGGAGTCTGGGCCGTGTCTCAGTCCCAGTGTGGCTGGTCATCCTCTCAGACCAGC
TAGGGATCGTCGCCTAGGTGAGCCGTTACCTCACCTACTAGCTAATCCCATCTGGGTTCATCCGATGGCGTGAGG
CCCGAAGGTCCCCCACTTTGCTCCTCAGAGGTCATGCGGTATTAGCCACCGTTTCCAGTGGTTATCCCCCTCCAT
CGGGCAGATCCCCAGACATTACTCACCCGTCCGCCGCTCGCCGGCCAGGAAGCAAGCTTCCTGCCGTTGCCGCTC
GACTTGCATGTGTTAGGCCTGCCGCCAGCGTTCAATCTGAGCCAGGATCAAACTCTAAGGGCGAATTCTGCAGAT
ATCCATCACACTGGCGGCCGCTCGAGCATGCATCTA

Accession No.	Species	Gene	Max scores	E-value	Identity (%)
CP016176.1	*X. hominickii* strain ANU1	16S rRNA	2750	0.0	99.54
AB507814.1	*X. hominickii* strain Sm1		2745	0.0	99.40
FJ515811.1	*X. hominickii* strain NkHk135		2717	0.0	99.60

[Colony # 3]

```
CCTACTTCTTTTGCAACCCACTCCCATGGTGTGACGGGCGGTGTGTACAAGGCCCGGGAACGTATTCACCGTAGC
ATTCTGATCTACGATTACTAGCGATTCCGACTTCATGGAGTCGAGTTGCAGACTCCAATCCGGACTACGACAGAC
TTTATGAGTTCCGCTTGCTCTCGCGAGGTCGCTTCTCTTTGTATCTGCCATTGTAGCACGTGTGTAGCCCTACTC
GTAAGGGCCATGATGACTTGACGTCATCCCCACCTTCCTCCGGTTTATCACCGGCAGTCTCCCTTGAGTTCCCAC
CATTACGTGCTGGCAACAAAGGATAAGGGTTGCGCTCGTTGCGGGACTTAACCCAACATTTCACAACACGAGCTG
ACGACAGCCATGCAGCACCTGTCTCAGCGTTCCCGAAGGCACTCCTCTATCTCTATAGGATTCGCTGGATGTCAA
GAGTAGGAAAGGGTCTTCGCGTTGCATCAAATTAGACCACATGCTCCACCGCTTGTGCGGAGCCCCGTCAATGCG
TTTGAGCTGTTATCATGCTGGGGGATTCCTCTAGCGGTGAATGATTAGAGTCGCGTCACAGGGGAGTAGCAATCC
TGCGTGAACCGACCAAAATGATCTACATATGTTTGCGGGAAATACTAGGAAGAGCGAACTACTCCTGTATTGCTC
ACCGAAACTTCCAGTGTCCGAAGCGTCTGCCAATCAAACAGGGGGGCCACCTATCGGCAAAATGTTATTCAGTCA
ATGTCATGTACCATATTTGCAGCGGTCACCCATTGAAAATTGCAACCTTCCCCGCTAGCCAATCAGTTTTAGATG
CAGTTCCCAGGTTAAGCCCGGGGATTTCACATCTAACTTAATTGACCGCCTGCGTGCGCTTTACGCCCAGTAATT
CCGATTAACGCTTGCACCCTCCGTATTACCGCGGCTGCTGGCACGGAGTTAGCCGGTGCTTCTTCTGCGGGTAAC
GTCAATCGTAAAGTCTGTTAAACTTCACGCCTTCCTCCCCGCTGAAAGTACTTTACAACCCGAAGGCCTTCTTCA
TACACGCGGCATGGCTGCATCAGGCTTGCGCCCATTGTGCAATATTCCCCACTGCTGCCTCCCGTAGGAGTCTGG
GCCGTGTCTCAGTCCCAGTGTGGCTGGTCATCCTCTCAGACCAGCTAGGGATCGTCGCCTAGGTGAGCCGTTACC
TCACCTACTAGCTAATCCCATCTGGGTTCATCCGATGGCGTGAGGCCCGAAGGTCCCCCACTTTGCTCCTCAGAG
GTCATGCGGTATTAGCCACCGTTTCCAGTGGTTATCCCCCTCCATCGGGCAGATCCCCAGACATTACTCACCCGT
CCGCCGCTCGCCGGCCAGGAAGCAAGCTTCCTGCCGTTGCCGCTCGACTTGCATGTGTTAGGCCTGCCGCCAGCG
TTCAATCTGAGCCAGGATCAAACTCTAAGGGCGAATTCTGCAGATATCCATCACACTGGCGGCCGCTCGAGCATG
CATCTA
```

Accession No.	Species	Gene	Max scores	E-value	Identity (%)
CP016176.1	*X. hominickii* strain ANU1	16S rRNA	2619	0.0	99.69
AB507814.1	*X. hominickii* strain Sm1		2603	0.0	99.54
DQ205449.1	*X. hominickii* strain KR05		2588	0.0	99.84

(B)

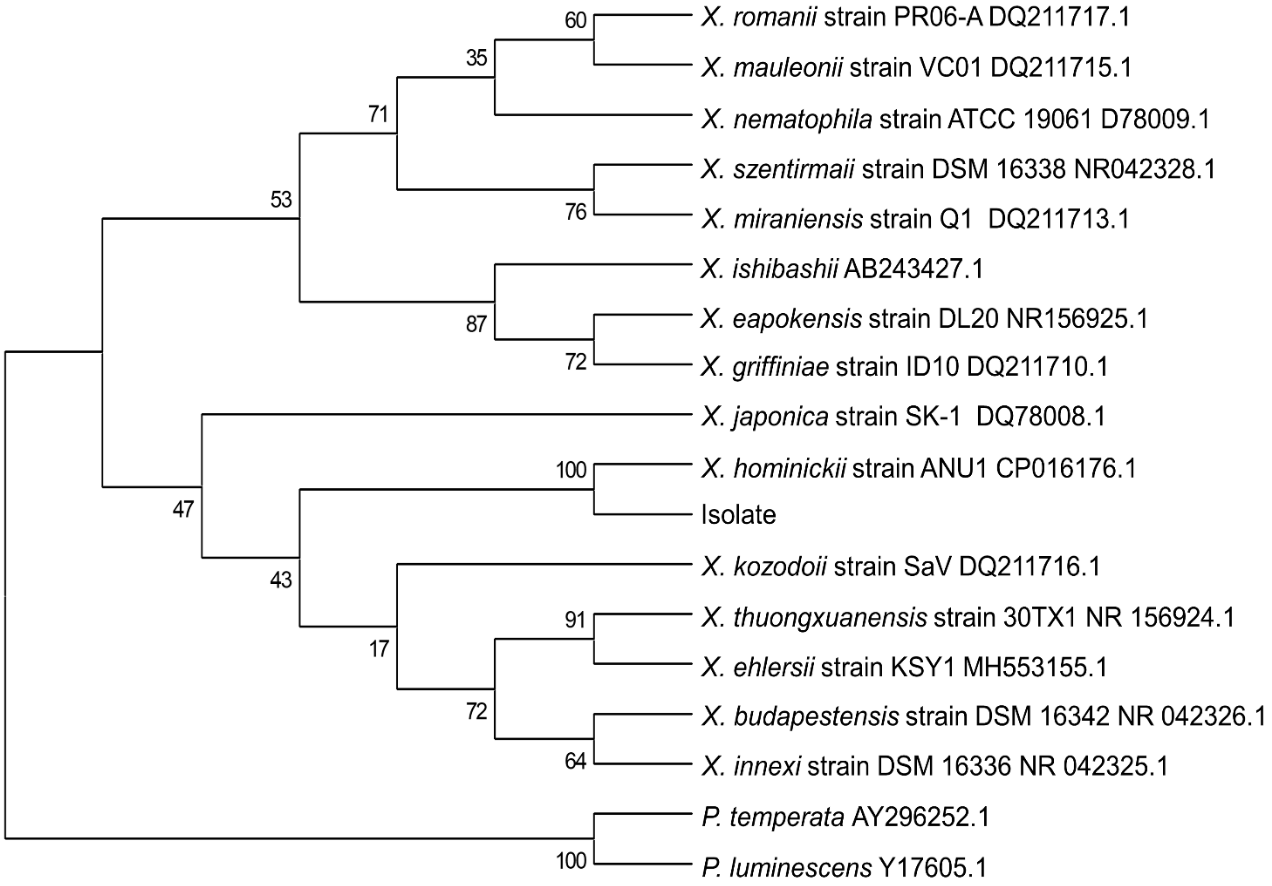

X. romanii strain PR06-A DQ211717.1
X. mauleonii strain VC01 DQ211715.1
X. nematophila strain ATCC 19061 D78009.1
X. szentirmaii strain DSM 16338 NR042328.1
X. miraniensis strain Q1 DQ211713.1
X. ishibashii AB243427.1
X. eapokensis strain DL20 NR156925.1
X. griffiniae strain ID10 DQ211710.1
X. japonica strain SK-1 DQ78008.1
X. hominickii strain ANU1 CP016176.1
Isolate
X. kozodoii strain SaV DQ211716.1
X. thuongxuanensis strain 30TX1 NR 156924.1
X. ehlersii strain KSY1 MH553155.1
X. budapestensis strain DSM 16342 NR 042326.1
X. innexi strain DSM 16336 NR 042325.1
P. temperata AY296252.1
P. luminescens Y17605.1
60
35
71
53
76
87
72
100
47
43
91
17
72
64
100

Fig. S1

(A)

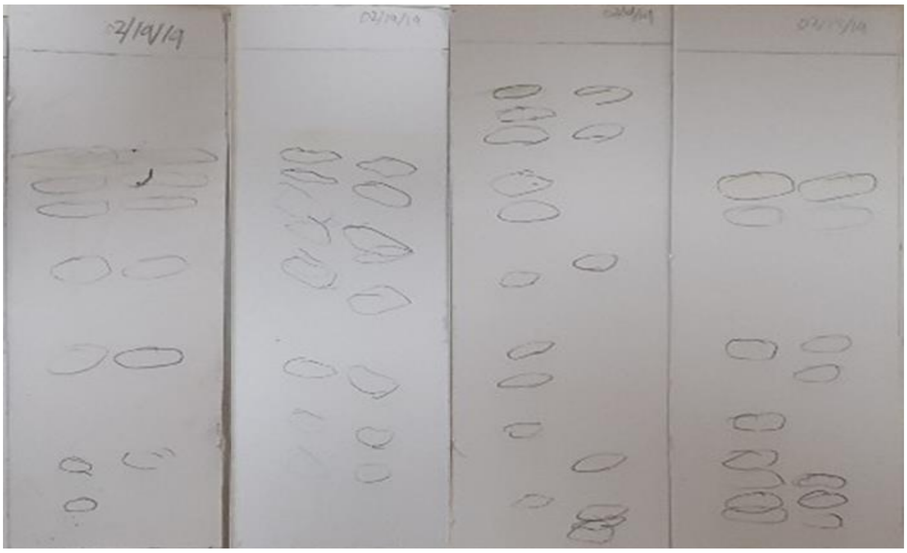

ANU DY / HEX   ANU DY / EAX   ANU DY / CX   ANU DY / BX

(B)

Rf	HEX		EAX		CX		BX	
	ANU	DY	ANU	DY	ANU	DY	ANU	DY
1	●	●	●	●	●	●	●	●
0.96					●			
0.94	●	●	●					
0.91			●	●	●	●	●	●
0.88	●	●						
0.85			●	●				
0.81					●			
0.75			●	●	●			
0.71	●	●	●	●				
0.61				●	●	●		
0.56								●
0.53							●	
0.49	●	●						●
0.44			●		●			
0.38					●			
0.32			●				●	
0.27				●	●			
0.21	●	●	●	●		●	●	●
0.12	●				●	●	●	●
0.07						●	●	●
0.04						●		
∑ 22	7	6	9	8	10	7	7	7

Fig. S2

(A) NRPS1

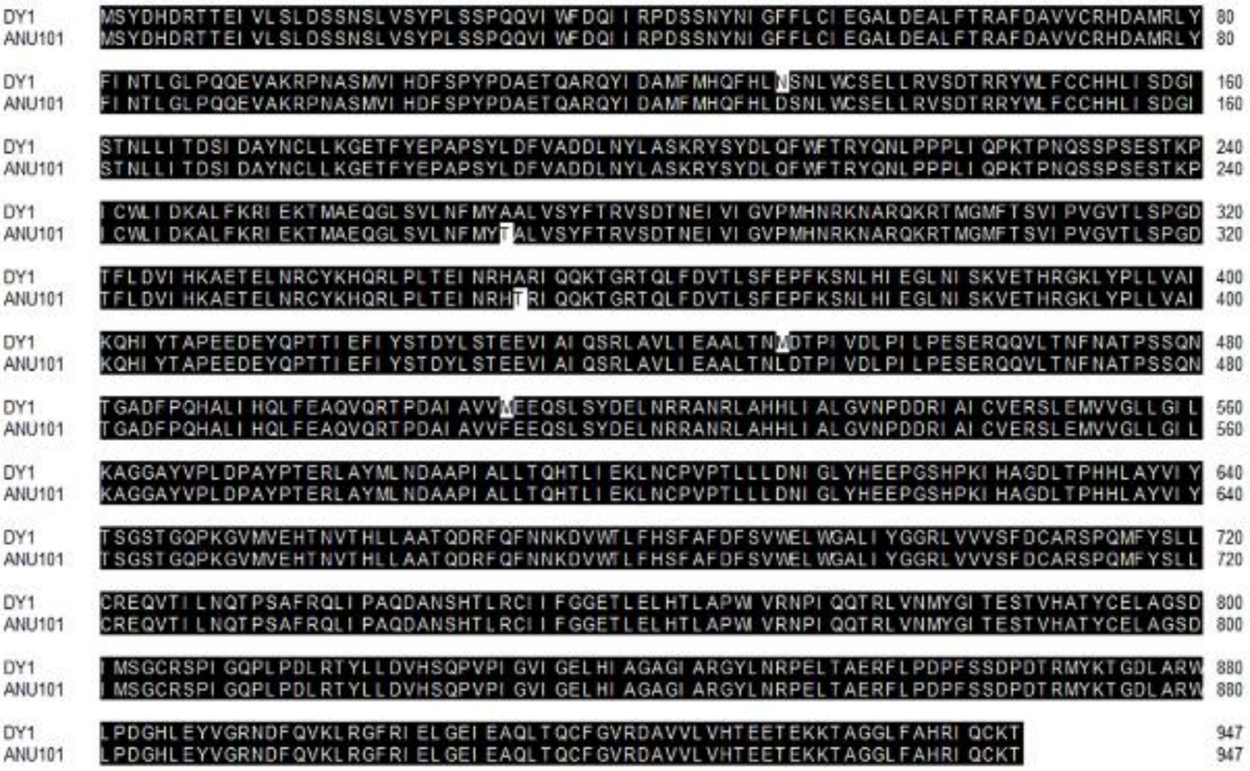
DY1 MSYDHDRTTEIVLSLDSSNSLVSYPLSSPQQVIWFDQIIRPDSSNYNIGFFLCIEGALDEALFTRAFDAVVCRHDAMRLY 80
ANU101 MSYDHDRTTEIVLSLDSSNSLVSYPLSSPQQVIWFDQIIRPDSSNYNIGFFLCIEGALDEALFTRAFDAVVCRHDAMRLY 80
DY1 FINTLGLPQQEVAKRPNASMVIHDFSPYPDAETQARQYIDAMFMHQFHLNSNLWCSELLRVSDTRRYWLFCCHHLISDGI 160
ANU101 FINTLGLPQQEVAKRPNASMVIHDFSPYPDAETQARQYIDAMFMHQFHLDSNLWCSELLRVSDTRRYWLFCCHHLISDGI 160
DY1 STNLLITDSIDAYNCLLKGETFYEPAPSYLDFVADDLNYLASKRYSYDLQFWFTRYQNLPPPLIQPKTPNQSSPSESTKP 240
ANU101 STNLLITDSIDAYNCLLKGETFYEPAPSYLDFVADDLNYLASKRYSYDLQFWFTRYQNLPPPLIQPKTPNQSSPSESTKP 240
DY1 ICWLIDKALFKRIEKTMAEQGLSVLNFMYAALVSYFTRVSDTNEIVIGVPMHNRKNARQKRTMGMFTSVIPVGVTLSPGD 320
ANU101 ICWLIDKALFKRIEKTMAEQGLSVLNFMYTALVSYFTRVSDTNEIVIGVPMHNRKNARQKRTMGMFTSVIPVGVTLSPGD 320
DY1 TFLDVIHKAETELNRCYKHQRLPLTEINRHARIQQKTGRTQLFDVTLSFEPFKSNLHIEGLNISKVETHRGKLYPLLVAI 400
ANU101 TFLDVIHKAETELNRCYKHQRLPLTEINRHTRIQQKTGRTQLFDVTLSFEPFKSNLHIEGLNISKVETHRGKLYPLLVAI 400
DY1 KQHIYTAPEEDEYQPTTIEFIYSTDYLSTEEVIAIQSRLAVLIEAALTNMDTPIVDLPILPESERQQVLTNFNATPSSQN 480
ANU101 KQHIYTAPEEDEYQPTTIEFIYSTDYLSTEEVIAIQSRLAVLIEAALTNLDTPIVDLPILPESERQQVLTNFNATPSSQN 480
DY1 TGADFPQHALIHQLFEAQVQRTPDAIAVVMEEQSLSYDELNRRANRLAHHLIALGVNPDDRIAICVERSLEMVVGLLGIL 560
ANU101 TGADFPQHALIHQLFEAQVQRTPDAIAVVFEEQSLSYDELNRRANRLAHHLIALGVNPDDRIAICVERSLEMVVGLLGIL 560
DY1 KAGGAYVPLDPAYPTERLAYMLNDAAPIALLTQHTLIEKLNCPVPTLLLDNIGLYHEEPGSHPKIHAGDLTPHHLAYVIY 640
ANU101 KAGGAYVPLDPAYPTERLAYMLNDAAPIALLTQHTLIEKLNCPVPTLLLDNIGLYHEEPGSHPKIHAGDLTPHHLAYVIY 640
DY1 TSGSTGQPKGVMVEHTNVTHLLAATQDRFQFNNKDVWTLFHSFAFDFSVWELWGALIYGGRLVVVSFDCARSPQMFYSLL 720
ANU101 TSGSTGQPKGVMVEHTNVTHLLAATQDRFQFNNKDVWTLFHSFAFDFSVWELWGALIYGGRLVVVSFDCARSPQMFYSLL 720
DY1 CREQVTILNQTPSAFRQLIPAQDANSHTLRCIIFGGETLELHTLAPWVRNPIQQTRLVNMYGITESTVHATYCELAGSD 800
ANU101 CREQVTILNQTPSAFRQLIPAQDANSHTLRCIIFGGETLELHTLAPWVRNPIQQTRLVNMYGITESTVHATYCELAGSD 800
DY1 IMSGCRSPIGQPLPDLRTYLLDVHSQPVPIGVIGELHIAGAGIARGYLNRPELTAERFLPDPFSSDPDTRMYKTGDLARW 880
ANU101 IMSGCRSPIGQPLPDLRTYLLDVHSQPVPIGVIGELHIAGAGIARGYLNRPELTAERFLPDPFSSDPDTRMYKTGDLARW 880
DY1 LPDGHLEYVGRNDFQVKLRGFRIELGEIEAQLTQCFGVRDAVVLVHTEETEKKTAGGLFAHRIQCKT 947
ANU101 LPDGHLEYVGRNDFQVKLRGFRIELGEIEAQLTQCFGVRDAVVLVHTEETEKKTAGGLFAHRIQCKT 947

(B) NRPS2

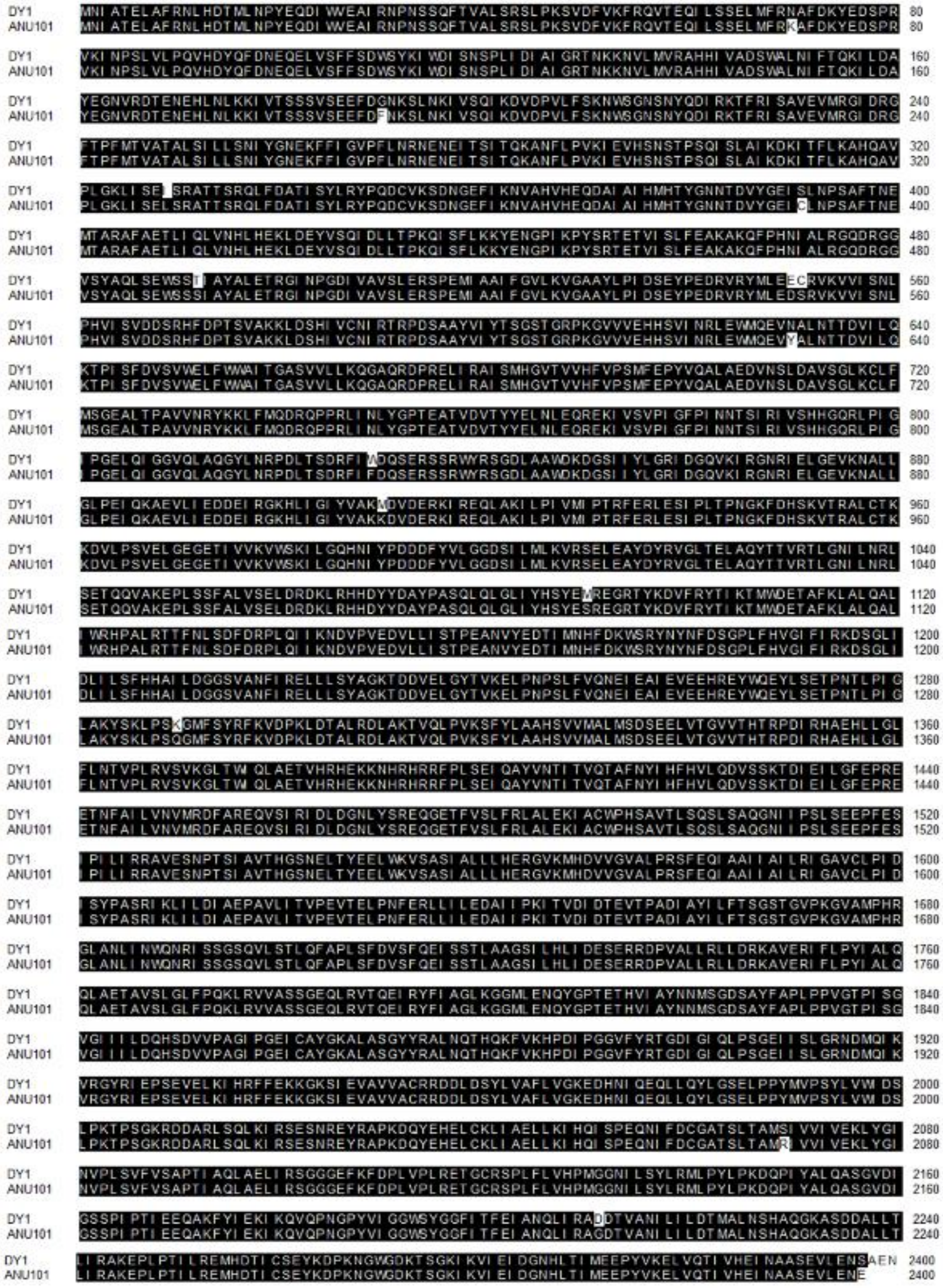

(C) NRPS3

```
DY1     MKKSQIVPLTQYQKEIYYEAQRNPESSQFTLSVMRILKENINLKKLFYSINNVLSYDVTCQLRIVEQAEGLSQQIQDITV  80
ANU101  MKKSQIVPLTQYQKEIYYEAQRNPESSQFTLSVMRILKENINLKKLFYSINNVLSYDVTCQLRIVEQAEGLSQQIQDITV  80

DY1     NQTEFIDAEGENVTVFFNKWSERIFNLDDNSLLEAALVRNLQNGCIAIFLRAHHIVCDGWGINQLAEKILQHYQNNDINQ  160
ANU101  NQTEFIDAEGENVTVFFNKWSERIFNLDDKSLLEAALVRNLQNGCIAIFLRAHHIVCDGWGINQLAEKILQHYQNNDINQ  160

DY1     VISYDNSEECISAITGQEIILSQPEIEQQITELAIFTQRHEPVFFNRGKKVNLSRFMHHRYILAKAEVEKAMSQETNPYQ  240
ANU101  VISYDNSEEYISAITGQEIILSQPEIEQQITELAIFTQRHEPVFFNRGKKVNLSRFMHHRYILAKAEVEKAMSQETNPYQ  240

DY1     VVMSAIAILLSRIHNNTQLTIGVPLLNRRINQLNVAGQWANTLPLAIEVKADDILLDLAARIKTDTTKLKTYEHIPLGRL  320
ANU101  VVMSAIAILLSRIHNNTQLTIGVPLLNRRINQLNVAGQWANTLPLAIEVKADDILLDLAARIKTDTTKLKTFEHIPLGRL  320

DY1     LKAIGTTKRQWFDVTISYNYLPIVDTFCYSYKNKDNYAAAHEDDAIAIHMSTYGDNNDVVIDITGAVDVFDEIYTFETFT  400
ANU101  LKAIGTTKRQWFDVTISYNYLPIVDTFCYSYKNKDNYAAAHEDDAIAIHMNTYGDNNDVVIDITGAVDVFDENYTFETFT  400

DY1     TSLERLIEQFIASPTQTVSEINLSSAEEMAHLVLFEQGPIAEFSKQDTLSTLFDTQISLHGKAVAIVNSDDQPTLTYTQF  480
ANU101  TSLERLIEQFIASPTQTVSEINLSSAEEMAQLVLFEQGPIAEFSKQDTLSTLFDTQISLHGKAVAIVNSDDQPTLTYTQF  480

DY1     QIRIDNMVTVLLKEGVRKGDIVAILMDRSTDMLTAIFAVLKAGAAYLPIDSNYPQERIDYMLNDSGVKVIISDKLNQSLL  560
ANU101  QIRIDNMVTVLLKEGVRKGDIVAILMDRSTDMLTAIFAVLKAGAAYLPIDSNYPQERIDYMLNDSGVKVIISDKLNQSLL  560

DY1     TESYIVIDGNNISAQENIKQPNLSTADSLAYMIYTSGSTGQPKGVMIDHYSVVNRIEWMQERYPLDNTDVILQKTPISFD  640
ANU101  TESYIVIDGNNISAQENIKQPNLSTADSLAYMIYTSGSTGQPKGVMIDHYSVVNRIEWMQERYPLDNTDVILQKTPISFD  640

DY1     VSVWELFWWSISGASVHLLPPGAHKDPLGIIKAINIHNVTTLHFVPSMLQPFLDILEAEPELVDKLTSLRRVFVSGEALP  720
ANU101  VSVWELFWWSISGASVHLLPPGAHKDPLEIIKAINIHNVTTLHFVPSMLQPFLDILEAEPELVDKLTSLRRVFVSGEALP  720

DY1     PTRVNQFRKIFSSFGLNAPALINLYGPTEATVDVSYFEFLPENNEVINRVPIGFPINNISLRIMSPHVTRQSIGIAGELQ  800
ANU101  PTRVNQFRKIFSSFGLNAPALINLYGPTEATVDVSYFEFLPENNEVINRVPIGFPINNISLRIMSPHGTRQSIGIAGELQ  800

DY1     ISGIGLARGYYNKSELTDEKFIWDREQRWYRTGDLARWLSDGSIEYLGRIDNQVKIRGNRIELGEVQNTLERIPGITQAE  880
ANU101  ISGIGLARGYYNKSELTDEKFIWDREQRWYRTGDLARWLSDGSIEYLGRIDNQVKIRGNRIELGEVQNTLERIPGITQAE  880

DY1     VLPQKNNNDDTYLCAYYVTNNLSFTSEKLRKILLEFLPDFMVPLKFFQVPSIPITPNGKVDREALRTFVKKEVSSKHDDA  960
ANU101  VLPQKNNNDDTYLCAYYVTNNLSFTSEKLRKILLEFLPDFMVPLKFFQVPSIPITPNGKVDREALRTFVKKEVSSKHDDA  960

DY1     RTETEYKLAEIWKRVLKIAQLSVQDNFYTLGGDSILMLKVRSEAEKAGMNVTLSELSQHLTIVELGQHIGISYKAQRTVE  1040
ANU101  RTETEYKLAEIWKRVLKIAQLSVQDNFYTLGGDSILMLKVRSEAEKAGMNVTLSELSQHLTIVELGQHIGISYKAQRTVE  1040

DY1     LSPLELIKEKERAKLSQYADAFPATQLQLGLIYHSQQSKGSATYKDVFRYTMKGEWKPDIFRKSAVATIQRHPVLRSHFN  1120
ANU101  LSPLELIKEKERAKLSQYADAFPATQLQLGLIYHSQQSKGSATYKDVFRYTMKGEWKPDIFRKSAVATIQRHPVLRSHFN  1120

DY1     LADFSEPLQVINHSIGFDHAIEIKDLSLLALVESEQYIKEYMKKRSCQDYHFDHSPLYHICLFKTIDTIEMIFSFHHAIL  1200
ANU101  LADFSEPLQVINHSIGFDHAIEIKDLSLLALVESEQYIKEYMKKRSCQDYHFDHSPLYHICLFKTIDTIEMIFSFHHAIL  1200

DY1     DGGSVANLIRELFSRYLNDGMYFSETDVLPSPALYIQDELHALQDQEHKTFWQEMFNGLPRTQFPSYRLYESLPKNRIFT  1280
ANU101  DGGSVANLIRELFSRYLNDGMYFSETDVLPSPALYIQDELHALQDQEHKTFWQEMFNGLPRTQFPSYRLYESLPKNRIFT  1280

DY1     KKALIPEEIEKNLKVLVQQENVSIKSVFFAAHCLTIAIVTNQDEIATGLVTHGRPDVQNSEQTLGLFLNTIPVRFSISDK  1360
ANU101  KKALIPEEIEKKLKVLVQQENVSIKSVFFAAHCLTIAIVTNQDEIATGLVTHGRPDVQNSEQTLGLFLNTIPVRFSISDK  1360

DY1     TWKECIHSVFSAGQRHAPYRKYPLNAIQQVAGEGARINSAFNYIHFHVLSEVFGSPDLSLIAFDPWEETNFEVLFNVMTD  1440
ANU101  TWKECIHSVFSAEQRHAPYRKYPLNAIQQVAGEGARINSAFNYIHFHVLSEVFGSPDLSLIAFDPWEETNFEVLFNVMTD  1440

DY1     FITGQNHLRCDFDGNIFSQVQADTYMDTYMTILNSMVSKGDQRAALSQPLTQRSKEIPSPEFISTIAMIMKNVLEQPDAI  1520
ANU101  FITGQNHLRCDFDGNIFSQVQADTYMDTYMTILNSMVSKGDQRAALSQPLTQRSKEIPSPEFISTIAMIMKNVQEQPDAI  1520

DY1     AIRYHKLQWSYEKLWLESGKIASALISEGVVQNTPVAIALERSPTLVASLIGVMRAGGVCLPLDLNYPTQRIEAMLEQAQ  1600
ANU101  AIRYHKLQWSYEKLWLESGKIASALISEGVVQNTPVAIALERSPTLVASLIGVMRAGGVCLPLDLNYPTQRIEAMLEQAQ  1600

DY1     PILAIVDSKYDNLLNIVTVPTIRVDTLINENTCVSDIEIAPEQLAYLLFTSGSTGKPKGVAMPHRSLSNWVTWQNETHTG  1680
ANU101  PILAIVDSKYDNLLNIVTVPTIRVDTLINENTCVSDIEIAPEQLAYLLFTSGSTGKPKGVAMPHRSLSNLVTWQNETHTG  1680

DY1     KNIRSTLQYAPLSFDVSFQEIFSTLTSGGELHLISENERRDPLQLLRIIDNYQTERIYLPYVALQQLAETAIALGTYPKN  1760
ANU101  KNIRSTLQYAPLSFDVSFQEIFSTLTSGGELHLISENERRDPLQLLRIIDNYQTERIYLPYVALQQLAETAIALGTYPKN  1760

DY1     LKTVISSGEQLRVTEDIRLFIGQINNGILENQYGPTETHVITAFTMQGNPKIFPALPPIGKGINNSDVVLLNSQLQEVAS  1840
ANU101  LKTVISSGEQLRVTEDIRLFIGQINNGILENQYGPTETHVITAFTMQGNPKIFPALPPIGKGINNSDVVLLNSQLQEVAS  1840

DY1     GEQGEIYVRGLPVALGYYRQPELTAERFIMFTDSDEPLYRTGDLGVKLKDDNILCLGRKDTQVKVRGYRIELAEVELAIL  1920
ANU101  GEQGEIYVRGLPVALGYYRQPELTAERFIMFTDSDEPLYRTGDLGVKLKDDNILCLGRKDTQVKVRGYRIELAEVELAIL  1920

DY1     STPGAEEILKDVAVVVQQRGENDSFLVAFLIGEKNEAFTVHVQQNLSETLPKHMLPSQIEWLESLPKTPSGKRDDAALRS  2000
ANU101  STPGAEEILKDVAVVVQQRGENDSFLVAFLIGEKNEAFTVHVQQNLSETLPKHMLPSQIEWLESLPKTPSGKRDDAALRS  2000

DY1     IRIIHGHNDVEDIAPRDNYEQVLCDLAADLLKIPSLSVHQNLFDIGGTSLTAMRMVVLVEKHFSTNIPLSLFVSNPTVAQ  2080
ANU101  IRIIHGHNDVEDIAPRDNYEQVLCDLAADLLKIPSLSVHQNLFDIGGTSLTAMRMVVLVEKHFSTNIPLSLFVSNPTVAQ  2080

DY1     LATYIRDKGGCPKFSPLVPMRTTGHKRPLFFVHPMGGNVLSYLRLVKYFPDDQPFYALQSHGVDAGSMPLTSVQEQAANY  2160
ANU101  LATYIRDKGGCPKFSPLVPMRTTGHKRPLFFVHPMGGNVLSYLRLVKYFPDDQPFYALQSHGVDAGSMPLTSVQEQAANY  2160

DY1     LEAIRQIQQHGQYSIGGWSYGGFVAFEIARQLKEHGEEVADLFVLDTVALNTESQGKANEEALLRWFFWELLWLNEGATL  2240
ANU101  LEAIRQIQQHGPYSIGGWSYGGFVAFEIARQLKEHGEEVADLFVLDTVALNTESQGKANEEALLRWFFWELLWLNEGATL  2240

DY1     PEQIVPEHITSLQERFEYITEHAIATGAIPQGSSRAVVQRLFDVYKTNWKAATDYAAYTADINVTLLHATRPLPEVLRSM  2320
ANU101  PEQIVPEHITSLQERFEYITEHAIATGAIPQGSSRAVVQRLFDVYKTNWKAATDYAAYTADINVTLLHATRPLPEVLRSM  2320

DY1     HDAIRSEYRDPKNGWESKTTGHINVIDVPGDHLEIMEVPYVAEVARVILRAMDESNPRKQVLLASESEIK  2390
ANU101  HDAIRSEYRDPKNGWESKTTGHINVIDVPGDHLEIMEVPYVAEVARVILRAMDEY-PRKQVLLASEEI   2390
```

(D) PKS1

```
DY1     MSESYLSLHDGFLHDKKVRGDIAIVGMASHFPDAPNLYKFWENIIEKKDSLIDITSKSEDEYWRKEDFYDPNPTVADKTY  80
ANU101  MSESYL-LHDGFLHDKKVRGDIAIVGMASHFPDAPNLYKFWENIIEKKDSLIDITSKSEDEYWRKEDFYDPNPTVADKTY  79

DY1     SHRAGFVPEIDFDPVEFKIPPAIVDSISTVQLFALYVAKQAMLDAGLIGQENHKVNRDRIGVILGGAGNGNTSFSLASRQ  160
ANU101  SHRAGFVPEIDFDPVEFKIPPAIVDSISTVQLFALYVAKQAMLDAGLIGQENHKVNRDRIGVILGGAGNGNTSFSLASRQ  159

DY1     QAPYLRRIILKAGLSEEVANEIIERTHGLYLEWNEDSFPGFLGNVACGRIASHFDLGGTSYMVDAACASSFAAIKAAIGE  240
ANU101  QAPYLRRIMMKAGLSEEVANEIIERTHGLYLEWNEDSFPGFLGNVACGRIASHFDLGGTSYMVDAACASSFAAIKAAIGE  239

DY1     LHSGSCDAVLTGGINLENSIFSFLCFSKTPALSKSNLSRPFDQSADGMMLGNGVGMLVLKRLEDAELDGDKIYAVIKSLE  320
ANU101  LHSGSCDAVLTGGINLENSIFSFLCFSKTPALSKSNLSRPFDQSADGMMLGDGVGMLVLKRLEDAELDGDKIYAVIKSLE  319

DY1     ASSDGRAKSIWAPRMEGQAKALRRAYDAAGLMPSDIQLVEAHGTGTASGDDTELKSLHTVFGEYSTPPKSVAIGSIKSQI  400
ANU101  ASSDGRAKSIFAPRMEGQAKALRRAYDAAGLMPSDIQLVEAHGTGTASGDDTELKSLHTVFGEYSTPPKSVAIGSIKSQI  399

DY1     GHTRCAAGAAGMIKVALALHHKVLPPTINVDKPTHQLLAENSPFYVNSEARPWLRSFNGTPRRAALSAFGFGGTNFHAIL  480
ANU101  GHTRCAAGAAAMIKVALALHHKVLPPTINVDKPTHQLLAENSPFYVNSEARPWLRSFNGTPRRAALSAFGFGGTNFHAIL  479

DY1     EEYEKQTHGRYRLNESPWIMLFKGADPAELLSQCEEALTCFSGHLPENAIRQHLEQQDIENLKPQQARILFVAQSVEQAV  560
ANU101  EEYEKQTHGRYRLNESPWIMLFKGADPAELLSQCEEALTCFSGHLPENAIRQHLEQQDIENLKPQQARILFVAQSVEQAV  559

DY1     ELLSIAIKQLRQNSASGWEHPRGIYYRPQGKALEGKIVALFPGQGSQYVNMARDIANDYPEMRQALETLDDVVISDLGHE  640
ANU101  ELLSIAIKQLRQNSASGWEHPRGIYYRPQGKALEGKIVALFPGQGSQYVNMARDIANDYPEMRQALETLDDVVISDLGHE  639

DY1     LSPVIYPIPAFSDEEREAQQQRLTDTANAQSALGAISVGYFNILKGMGFTPDFVAGHSYGEVTALWAAGVLSDKDFYRVS  720
ANU101  LSPVIYPIPAFSDEEREAQQQRLTDTANAQPALGAISVGYFNILKGMGFTPDFVAGHSYGEVTALWAAGVLSDKDFYRVS  719

DY1     LIRGRAAASASALRSTNTGGTNTDAGAMLAASLAYEQRAEILARYPDLVIANDNSAQQVVFGGATSLINQLHDELKKHAV  800
ANU101  LIRGRAAASASALRSTNTGGTNTDAGAMLAASLAYEQRAEILARYPDLVIANDNSAQQVVFGGATSLINQLHDELKKHAV  799

DY1     HCRILPVSAAFHTKFIQPAYPLYRECLADINFQSPQCRLFSSATTEPYEHASQAIRELLAEQLIKPVKFRQAIEAIYQQG  880
ANU101  HCRILPVSAAFHTKFIQPAYPLYRECLADINFQSPQCRLFSSATTEPYEHASQAIRELLAEQLIKPVKFRQTIEAIYQQG  879

DY1     GRLFVEVGPKGVLGKLVADILQHREHTVISVNPNDKGEERLQFARAQAQLLAEGIKLREINQHVRPQPMTEDKSKRLTFK  960
ANU101  GRLFVEVGPKGVLGKLVADILQHREHTVISVNPNDKGEERLQFARAQAQLLAEGIKLREINQHVRPQPMTEDKSKRLTFK  959

DY1     LTGGFFLSAKNQARRQHALRDDDRTTVEQLIAESVSASVPSAPILQPRNTTESKNEQAVESVVEKAVEEKEVEKEEVEKE  1040
ANU101  LTGGFFLSAKNQARRQHALRDDDRTTVEQFIAESVSASVPSAPILQPRNTTESKNEQAVESVVEKAVEEKEVEKEEVEKE  1039

DY1     VKTEIKTEAKNNQVASTQFIQQNMLTIIQGRDNPMEQNNQLSENLDVNMVNVKTLNGLLQAQQVMSQLHQQFQANQQEYI  1120
ANU101  VKTEIKTEAKNNQVASTQFIQQNMLTIIQRRDNPMEQNNQLSENLDVNMVNVKTLNGLLQAQQVMSQLHQQFQANQQEYI  1119

DY1     QLLAMLLDKQYNLLESCKDHQNLPTMLSSLSQSVQLLDKNLELYHTNHERYFAVQQSLFQSGQIASVSSLTQSVVGYSAT  1200
ANU101  QLLAMLLDKQYNLLESCKDHQNLPTMLSSLSQSVQLLDKNLELYHTNHERYFAVQQSLFQSGQIASVSSLTQSVVGYSAT  1199

DY1     NVAPSAPMTVIEKVAPTVSVEPAPVSVSTVQSPTIQSPTVASPKVEPAKNPEKTETMMEITPLRSTIPSMPKTEEILATP  1280
ANU101  NVAPSAPMTVIEKVAPTVSVEPAPVSVSTVQSPTIQSPTVASPKVEPAKKPEKTETMMEITPLRSTIPSMPKTEEILATP  1279

DY1     TMPSETVAAEPIKPAPVEAEVKVQAKVQTKVLDPEVEKQIKRFEQITEESIISQLVAIVSERTGYPEDMITPEMDLEADL  1360
ANU101  TMPSETVAAEPIKPAPVEAEVKVQAKVQTKVLDPEVEKQIKRFEQITEESIISQLVAIVSERTGYPEDMITPEMDLEADL  1359

DY1     GIDSIKRLEIFGAMFDAFSADAGIYHDSSKNKDLDTFDIDALSNISKMAVFFKQMINETIEDLLNQGGDKTGSETLSSAT  1440
ANU101  GIDSIKRLEIFGAMFDAFSADAGIYHDSSKNKDLDTFDIDALSNISKMAVFFKQMINETIEDLLNQGGDKTGSETLSSAT  1439

DY1     EVVEERHSYEDSKGHAADSGNSLGKLQTLGFFTTTVGSHGADSVKKPLAEPQLSVSPAVQPALNNQTVLNEQAVLNQAVL  1520
ANU101  EVVEERHSYEDSKGHAADSGNSLGKLQTLGFFTTTVGSHGADSVKKPLAEPQLSVSPAVQPALNNQTVLNEQAVLNQAVL  1519

DY1     NEEASFADSPVSRFAVVKHSLPKPECLPGVFLSSKRWLVVDEGTNSADHLVAALRQQGQQVAVLTLGQNSIAMGDEAALS  1600
ANU101  NEEASFADSPVSRFAVVKHSLPKPECLPGVFLSSKRWLVVDEGTNSADHLVAALRQQGQQVAVLTLGQNSIAMGDEAALS  1599

DY1     ATLTDIEQQQGTIEGVIYLQAPKLPVPEKSVNAVAEVFNERDYRSVETTFLLAKHLQGSLNHASPQVGYFIVVMRGDGEL  1680
ANU101  ATLTDIEQQQGTIEGVIYLQAPKLPVPEKSVNAVAEVFNERDYRSVETTFLLAKHLQGSLNHASPQVGYFIVVMRGDGEL  1679

DY1     LTSGREYLPIVSAGVTGLTKSLNIEWGNVSCRTIDIDVRIKDNDVAQIVMEELQDHRTDLAEIGRSVNGERMTLALTEEK  1760
ANU101  LTSGREYLPIVSAGVTGLTKSLNIEWGNVFCRTIDIDVRIKDNDVAQIVMEELQDHRTDLAEIGRSVNGERMTLALTEEK  1759

DY1     VLSASSVNQVNTSDVLVVTGGARGITAQCVIELAKQRQAAFILLGRTDITVPLPEWAEGKTTLDERKMAAIAYLQAQGVQ  1840
ANU101  VLSASSVNQVNTSDVLVVTGGARGITAQCVIELAKQRQAAFILLGRTDITVPLPEWAEGKTTLDERKMAAIAYLQAQGVQ  1839

DY1     PTPVKINSMLDPLSHIAEINATLQAIKQAGGRAIYLHCDITNAEQVKMALSKAQQQLGQITGLIHGAGNLADKRIEKKTL  1920
ANU101  PTPVKINSMLDPLSHIAEINATLQAIKQAGGRAIYLHCDITNAEQVKMALSKAQQQLGQITGLIHGAGNLADKRIEKKTL  1919

DY1     ADLHSVFNAKVKGLENLCRELDCTSLRHIMLFSSVAGFFGNAGQTDYSLANEVLNKFVYLPFKGKNSQIVRSVNWGPWDG  2000
ANU101  ADLHSVFNAKVKGLENLCRELDCTSLRHIMLFSSVAGFFGNAGQTDYSLANEVLNKFVYLPFKGKNSQIVRSVNWGPWDG  1999

DY1     GMVSDVLKRAYEAQNMVIIPLEEGIQRFVREFRDPRSCQITLGGETYKAPKKTNISRNNNRYHSPS                2066
ANU101  GMVSDVLKRAYEAQNMVIIPLEEGIQRFVREFRDPRSCQITLGGETYKAPKKTNISRNNNRYHSPS                2066
```

(E) PKS2

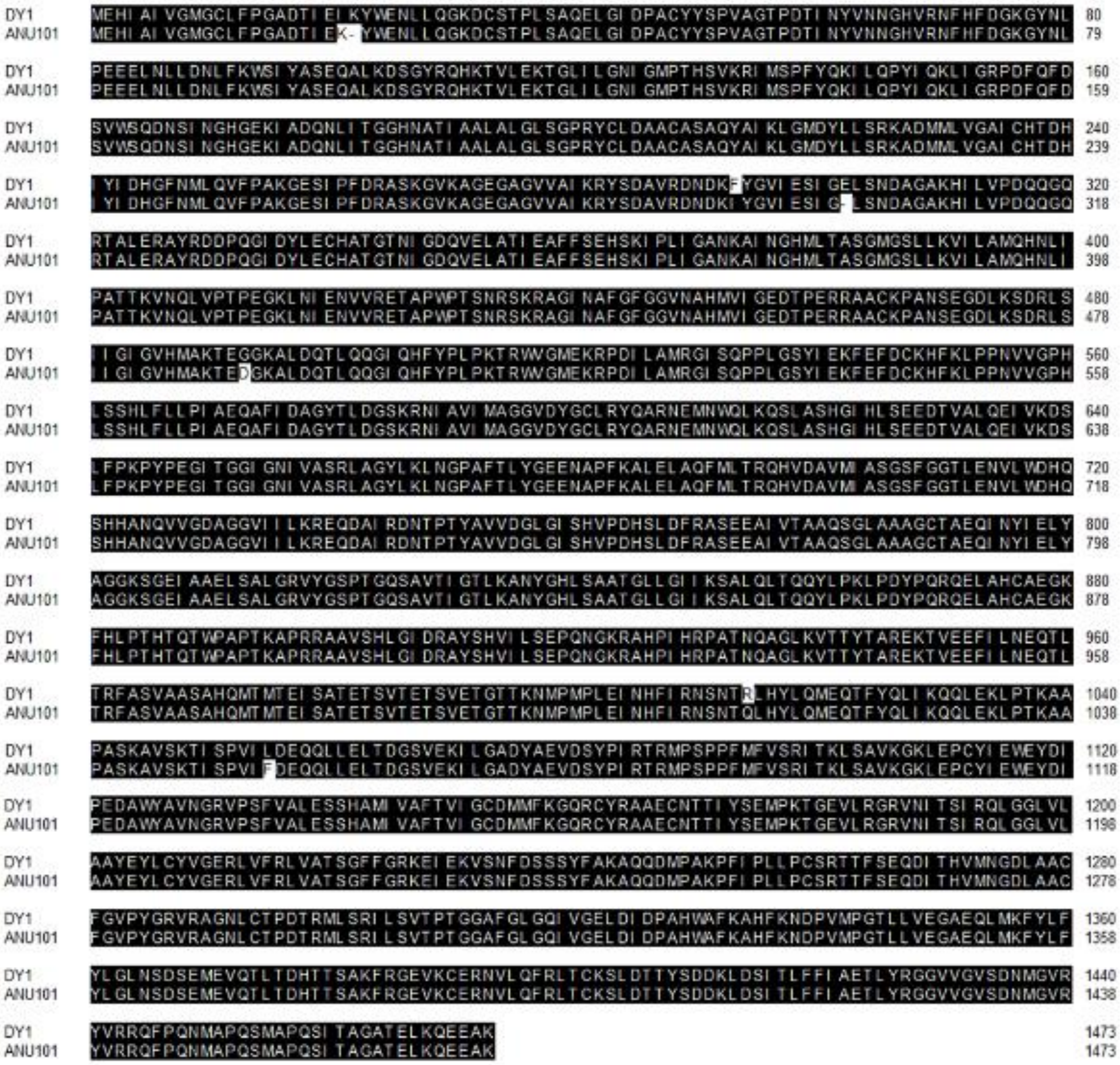

(F) PKS3

```
DY1     MNNAEL NKVQAMGSDESMHGFEMNDFEMNGFEI AI I GMAGGQYSGANDI EAFWQNI AAGVESVRPL SDAEL DAAGI SI EAR   80
ANU101  MNNAEL NKVQAMGSDESMHGFEMNDFEMNGFEI AI I GMAGGQYSGANDI EAFWQNI AAGVESVRPL SDAEL DAAGI SI EAR   80

DY1     SRSDFI RNVSVL DNPEDFDAEFFGYSPREAQVI DPQQRLLLETSWL ALENAGYVSDKYPGTI GVFGSSSSSSYLLHYLLK   160
ANU101  SRSDFI RNVSVL DNPEDFDAEFFGYSPREAQVI DPQQRLLLETSWL ALENAGYVSDKYPGTI GVFGSSSSSSYLLHYLLK   160

DY1     YPSAMQHFLSDML LFGNNNDLLSTRVAYKL NL RGPSLTI GSACSSSL VAL HYACQSLLAGECDMAL GAGVHI GFPSHSGY   240
ANU101  YPSAMQHFLSDML LFGNNNDLLSTRVAYKL NL RGPSLTI GSACSSSL VAL HYACQSLLAGECDMAL GAGVHI GFPSHSGY   240

DY1     HFDSSGI LSHDGHCRTFDADSSGTLSGDGVGVVVLKRL QDAL DSGDNI YAVI KGSAVNNDGAGKI GFTAPSVAGQTVVLQ   320
ANU101  HFDSSGI LSHDGHCRTFDADSSGTLSGDGVGVVVLKRL QDAL DSGDNI YAVI KGSAVNNDGAGKI GFTAPSVAGQTVVLQ   320

DY1     RALLAAEVSAESVSYI EAHGTATHL GDPMEMQALLNVYDQDAVAVREGL AQEKSAQQRCAVASVKANVGHTDSAAGVTGV   400
ANU101  RALLAAEVSAESVSYI EAHGTATHL GDPMEMQALLNVYDQDAVAVREGL AQEKSAQQRCAVASVKANVGHTDSAAGVTGV   400

DY1     MKVAQML THRYLPPAVNFTRL NPEI NLTGSRLYI PTEGQEWQSQSI RRAGVSSF GVGGTNAHMI LQETAYTPSSGPARPQ   480
ANU101  MKVAQML THRYLPPAVNFTRL NPEI NLTGSRLYI PTEGQEWQSQSI RRAGVSSF GVGGTNAHMI LQETAYTPSSGPARPQ   480

DY1     QLLCFSAKSPAAL QANL KKFRHWL EKSAQL PL ADI AYTLHNVGRSEMEYRHNL VCDDHSQAI AAL DNHL SQFSQGTI ART   560
ANU101  QLLCFSAKSPAAL QANL KKFRHWL EKSAQL PL ADI AYTLH-VGRSEMEYRHNL VCDDHSQAI AAL DNHL SQFSQGTI ART   559

DY1     TASSVVFMFSGQGSQYSGMMRGYYDQI SSNQVNNDHKTRFKAVVDECAEI LLPLLGRDI RPLI FDLTERGEESALYQTRY   640
ANU101  TASSVVFMFSGQGSQYSGMMRGYYDQI SSNQVNNDHKTRFKAVVDECAEI LLPLLGRDI RPLI FDLTERGEESALYQTRY   639

DY1     TQPALFVVEYSLRQLLMEWGI HPTACI GHSI GEYVAACLAGVFTLEDALTI VVRRAELMNRMPTGKML AVGL NTEAL QGY   720
ANU101  TQPALFVVEYSLSQLLMEWGI HPTACI GHSI GEYVAACLAGVFTLEDALTI VVRRAELMNRMPTGKML AVGL NTEAL QGY   719

DY1     LTDGVTLAADNAPEL CVVSGETEAVLALETRLTAEGI DARRLHTSHAFHSAMMDECLESFRQAFNEI TLSPPKLPFI SCI   800
ANU101  LTDGVTLAADNAPEL CVVSGETEAVLALETRLTAEGI DARRLHTSHAFHSAMMDECLESFRQAFNEI TLSPPKLPFI SCI   799

DY1     TGEW SAEQATSPDYWWRQL REPVAFKQGI ACLLSANTVSSSAVSSNSKLLLEVGPGSVL SGL ARMQMTGVSAHQQVFSS   880
ANU101  TGEW SAEQATSPDYWWRQL REPVAFKQGI ACLLSANTVSSSAVSSNSKLLLEVGPGSVL SGL ARMQMTGVSAHQQVFSS   879

DY1     TRDI KQDI PDRQHLLSVL GKL WQQGASVDWTAL YRQEKRRRL PL PGYVF QHQRYSLESLI GNHSEL STRPEL RSSKRL PL   960
ANU101  TRDI KQDI PDRQHLLSVL GKL WQQGASVDWTAL YRQEKRRRL PL PGYVF QHQRYSLESLI GNHSEL STRPEL RSSKRL PL   959

DY1     EQWSHQI RWKQMAASSVGNI SLHGQVFLI FMDRL GVGKRLTTQL EQL GGEVWKVVQGRHFNANPQL RRI MI NPQQGEDYQ   1040
ANU101  EQWSHQI RWKQMAASSVGNI SLHGQVFLI FMDRL GVGKRLTTQL EQL GGEVWKVVQGRHFNANPQL RRI MI NPQQGEDYQ   1039

DY1     ALAALFKRQKI QI NNI VHAWSI TRSSTI SLQTAFTNYESLLNHI ARQLAVGRI ENQHDTSLDTSQFKI NI LSNRLHAI SE   1120
ANU101  ALAALFKRQKI QI NNI VHAWSI TRSSTI SLQTAFTNYESLL-HI ARQLAVGRI ENQHDTSLDTSQFKI NI LSNRLHAI SE   1118

DY1     YDRNDPAKALLI GPCRVI PQEFEQVQCKSI DI ALSANRWSLPLLSPVSSNML VSALLAEI LGAQPGEDPKENNI VALRGR   1200
ANU101  YDRNDPAKALLI GPCRVI PQEFEQVQCKSI DI ALSANRWSLPLLSPVSSNML VSALLAEI LG-QPGEDPKENNI VALRGR   1197

DY1     QRYI PLI ETAI LPALI DDSSNAGSEVTRSPLFRQGGNYVI TGGFGGI GATI ATHLASQYQPHLI FI SRSELPPKNEWSSW   1280
ANU101  QRYI PLI ETAI LPALI DDSSNAGSEVTRSPLFRQGGNYVI TGGFGGI GATI ATHLASQYQPHLI FI SRSELPPKNEWSSW   1277

DY1     I ENHDSNNKRSRHI RLLQQL QQMGASVSVI SADLADARRI NKALTDI VI QYGQI HGI FHSAGI ADAGLI QNRSFEDSLQV   1360
ANU101  I ENHDSNNKRSRHI RLLQQL QQMGASVSVI SADLADARRI NKALTDI VI QYGQI HGI FHSAGI ADAGLI QNRSFEDSLQV   1357

DY1     FKSKVI GTQVLDKWLKRHPL DFFVL CSSLASTVGSI GQVAYCAANAFEDAYALLHHQYRSNKKEQTRYLAI GWDSWRDVG   1440
ANU101  FKSKVI GTQVLDNWLKRHPL DFFVL CSSLASTVGSI GQVAYCAANAFEDAYALLHHQYRSNKKEQTRYLAI GWDSWRDVG   1437

DY1     MAVDSMSQWYQNDKEQESI KHGI LPDEGCQLLEALLAHNDSVYAI STRGLPL PDVNI SEEQDERAVTATMVKTSTQKSGQ   1520
ANU101  MAVDSMSQWYQNDKEQESI KHGI LPDEGCQLLEALLAHNDSVYAI STRGLPL PDVNI SEEQDERAVTATMVKTSTQKSGQ   1517

DY1     ASSTQQSI QQI NEQTLYPRPVL SVDYQPPRNQTETI I ADI WHEKMGI GPL GVFDDFFEL NGHSMMAVQI I AKI KQRL QVA   1600
ANU101  ASSTQQSI QQI NEQTLYPRPVL SVDYQPPRNQTETI I ADI WHEKMGI GPL GVFDDFFEL NGHSMMAVQI I AKI KQRL QVA   1597

DY1     FPVGFI YEYATI AQL AEQVERRNKPQENHEVDMPEGGK   1638
ANU101  FPVGFI YEYATI AQL AEQVERRNKPQENHEVDMPEGGK   1638
```

(G) PKS4

```
DY1     MQKYALPDEMAAI EI NLPGELDVLTLVKMPVPKLEI GEI LVKVAVAGVNRPDVL QRRGHYPPPQGASDMPGLEI SGTVVA   80
ANU101  MQKYALPDEMAAI EI NLPGELDVLTLVKMPVPKLEI GEI LVKVAVAGVNRPDVL QRRGHYPPPQGASDI PGLEI SGTVVA   80

DY1     VGPQVKHYTVGDQVCALVAGGGYAEYCNVHESNVLPVPSGYSLLEAAALPETFFTVWWNLFQRGKLKAGETVLI HGGTSG   160
ANU101  VGPQVKHYTVGDQVCALVAGGGYAEYCNVHESNVLPVPSGYSLLEAAALPETFFTVWWNLFQRGKLKAGETVLI HGGTSG   160

DY1     I GTTATLLAKAFNARVI TTVGSEEKRQASLALGADFSI NYRNDDFVHKLHQI TNGHGADVKI VDLI AGDYVEKNFQAAAI   240
ANU101  I GTTATLLAKAFNARVI TTVGSEEKRQASLALGADFSI NYRNDDFVHKLHQI TNGHGADV-I VDLI AGDYVEKNFQAAAI   239

DY1     EGRI I QVGLQHGKVKELDLMPLLKKRLI I TGSTLRSRSVEDKAI I ASELREKI WPLLEQGVI RPQI FKTFSLDQASEAHA   320
ANU101  EGRI I QVGLQHGKVKELDLMPLLKKRLI I TGSTLRSRSVEDKAI I ASELREKI WPLLEQGVI RPQI FKTFSLDQASEAHA   319

DY1     LMENSSHI GKI MLMV   335
ANU101  LMENSSHI GKI MLMV   335
```

Fig. S3

(A)

```
Lrp1        ATGATTGATAATAAGAAGCGTCCAGGAAAAGATCTTGATCGCATAGACCGAAATATCCTT  60
Lrp2        ------------------------------ATGCTAGACAAAATAGACCGTAAGCTACTG  30
                                             ** **    ******** **  * **

Lrp1        AATGAGTTGCAAAAAGACGGCCGTATTTCTAATGTGGAATTATCCAAGCGTGTTGGTTTG  120
Lrp2        GAACTGCTCCAACAGGATTGTAGTCTATCTCTGAATACGCTGGCCGAAGCTGTTAATTTG  90
             *   * * *** * **  *  ** * ***          *  ** *   ****  ****

Lrp1        TCACCAACGCCTTGTCTGGAGCGTGTACGTCGCTTAGAGCGTCAGGGTTTCATTTCTGGC  180
Lrp2        ACTTCTACACCCTGCTGGAAACGTCTAAAAAGACTTGAAGATGAAGGATATATTGTTGGA  150
             *  * ** ** **   * * *** **    *  * **   * * ** *  ***  ***

Lrp1        TATACAGCTTTGCTGAATCCTCATTATTTGGACGCATCATTGCTGGTGT------TTGTT  234
Lrp2        AAAGTAGCCTTGCTAAATGGTCCAAAACTTGGGCTTAGTCTGACGGTGATAGTGATGATT  210
             *   *** ***** ***  **   *  * *         **  ****       *  **

Lrp1        GAAATTACGCTGAATCGTGGTGCAGCGGACGTATTTGAACAATTCAATACAGCAGTCCAG  294
Lrp2        AAAACTCAGCAGCACAGTAGTGAAT------GGTATGAACAGTTTGTTGCATTCATAAAA  264
             *** *  ** * *  ** *** *         * ****** **   * **    *  *

Lrp1        AAACTGGAAGAAATCCAAGAATGCCATTTAGTTTCTGGTGATTTTGACTATTTGCTGAAA  354
Lrp2        GAGATGCCGGAAGTTTTAACATTTTATCGAATGGCAGGAGAATATGATTATCTAATGCAT  324
             *  **   *** *   *  **   **  * *  * ** ** * *** *** *  ** *

Lrp1        ACGCGTGTGCCAGATATGTCTGC---------ATACCGTAAACTACTGGGTGAAACGTTG  405
Lrp2        GTAGAAGTTGTTGATATGAAAAGTTATGATCGCTTCTATAAACGCATGGTAAATGG----  380
                  **    ******               * *  *****   ***    *

Lrp1        CTGCGTCTCCCGGGTGTTAACGATACTCGTACTTATGTTGTTATGGAAGAAGTGAAACAG  465
Lrp2        -GGTTTCTGGCT----TGATTGATGTAACCTCCAACTTTGCAATGGAGAAAATAAAATAT  435
              *  ***  *     * *  ***       *  *  ***  *****  ** * *** *

Lrp1        AGTAATCGTTTGGTGAT-TAAGACTCGTTAA-----  495
Lrp2        ACAACGGCGTTGCCAATACCAGATTCAGTAATATGA  471
            *  *     ***   **   *** **  ***
```

(B)

```
Lrp1        MIDNKKRPGKDLDRIDRNILNELQKDGRISNVELSKRVGLSPTPCLERVRRLERQGFISG  60
Lrp2        ----------MLDKIDRKLLELLQQDCSLSLNTLAEAVNLTSTPCWKRLKRLEDEGYIVG  50
                       **:***::*: **:*  :*   *:: *.*: *** :*::*** :*:* *

Lrp1        YTALLNPHYLDASLLVFVEITLNRGAADVFEQFNTAVQKLEEIQECHLVSGDFDYLLKTR  120
Lrp2        KVALLNGPKLGLSLTVIVMIKTQQHSSEWYEQFVAFIKEMPEVLTFYRMAGEYDYLMHVE  110
             .****   *. ** *:* *. :: ::: :*** : :::: *:    : ::*::***::..

Lrp1        VPDMSAYRKLLGETLLRLPGVNDTRTYVVMEEVKQSNRLVIKTR--        164
Lrp2        VVDMKSYDRFYKRMVNGVSGLIDVTSNFAMEKIKYTTALPIPDSVI        156
            * **.:* ::  . :  : *: *. : ..**::* :. * *
```

Fig. S4

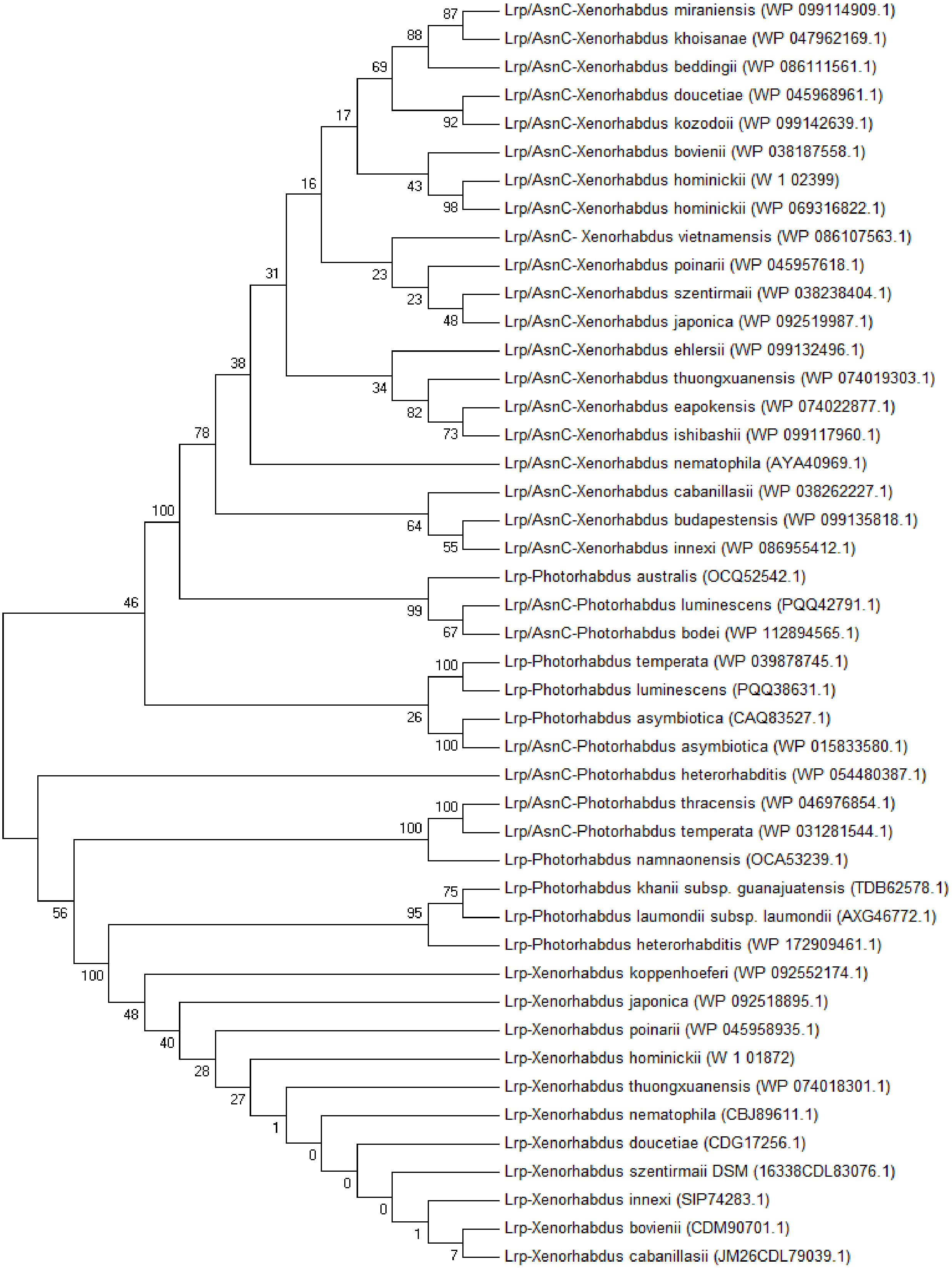
Lrp/AsnC-Xenorhabdus miraniensis (WP 099114909.1)
Lrp/AsnC-Xenorhabdus khoisanae (WP 047962169.1)
Lrp/AsnC-Xenorhabdus beddingii (WP 086111561.1)
Lrp/AsnC-Xenorhabdus doucetiae (WP 045968961.1)
Lrp/AsnC-Xenorhabdus kozodoii (WP 099142639.1)
Lrp/AsnC-Xenorhabdus bovienii (WP 038187558.1)
Lrp/AsnC-Xenorhabdus hominickii (W 1 02399)
Lrp/AsnC-Xenorhabdus hominickii (WP 069316822.1)
Lrp/AsnC- Xenorhabdus vietnamensis (WP 086107563.1)
Lrp/AsnC-Xenorhabdus poinarii (WP 045957618.1)
Lrp/AsnC-Xenorhabdus szentirmaii (WP 038238404.1)
Lrp/AsnC-Xenorhabdus japonica (WP 092519987.1)
Lrp/AsnC-Xenorhabdus ehlersii (WP 099132496.1)
Lrp/AsnC-Xenorhabdus thuongxuanensis (WP 074019303.1)
Lrp/AsnC-Xenorhabdus eapokensis (WP 074022877.1)
Lrp/AsnC-Xenorhabdus ishibashii (WP 099117960.1)
Lrp/AsnC-Xenorhabdus nematophila (AYA40969.1)
Lrp/AsnC-Xenorhabdus cabanillasii (WP 038262227.1)
Lrp/AsnC-Xenorhabdus budapestensis (WP 099135818.1)
Lrp/AsnC-Xenorhabdus innexi (WP 086955412.1)
Lrp-Photorhabdus australis (OCQ52542.1)
Lrp/AsnC-Photorhabdus luminescens (PQQ42791.1)
Lrp/AsnC-Photorhabdus bodei (WP 112894565.1)
Lrp-Photorhabdus temperata (WP 039878745.1)
Lrp-Photorhabdus luminescens (PQQ38631.1)
Lrp-Photorhabdus asymbiotica (CAQ83527.1)
Lrp/AsnC-Photorhabdus asymbiotica (WP 015833580.1)
Lrp/AsnC-Photorhabdus heterorhabditis (WP 054480387.1)
Lrp/AsnC-Photorhabdus thracensis (WP 046976854.1)
Lrp/AsnC-Photorhabdus temperata (WP 031281544.1)
Lrp-Photorhabdus namnaonensis (OCA53239.1)
Lrp-Photorhabdus khanii subsp. guanajuatensis (TDB62578.1)
Lrp-Photorhabdus laumondii subsp. laumondii (AXG46772.1)
Lrp-Photorhabdus heterorhabditis (WP 172909461.1)
Lrp-Xenorhabdus koppenhoeferi (WP 092552174.1)
Lrp-Xenorhabdus japonica (WP 092518895.1)
Lrp-Xenorhabdus poinarii (WP 045958935.1)
Lrp-Xenorhabdus hominickii (W 1 01872)
Lrp-Xenorhabdus thuongxuanensis (WP 074018301.1)
Lrp-Xenorhabdus nematophila (CBJ89611.1)
Lrp-Xenorhabdus doucetiae (CDG17256.1)
Lrp-Xenorhabdus szentirmaii DSM (16338CDL83076.1)
Lrp-Xenorhabdus innexi (SIP74283.1)
Lrp-Xenorhabdus bovienii (CDM90701.1)
Lrp-Xenorhabdus cabanillasii (JM26CDL79039.1)
87
88
69
17
92
16
43
98
31
23
23
48
38
34
82
73
78
100
64
55
46
99
67
100
26
100
100
100
75
95
56
100
48
40
28
27
1
0
0
0
1
7

Fig. S5
